# Erythrocyte Membrane Biophysical Changes Mediated by Pooled Immunoglobulin G and Hematin: Electrokinetic and Lipid Peroxidation Studies

**DOI:** 10.3390/membranes13030281

**Published:** 2023-02-27

**Authors:** Virjinia Doltchinkova, Meglena Kitanova, Rumen Nikolov, Angelina Stoyanova-Ivanova, Ognyan Petkov, Yoana Dikova, Victoria Vitkova

**Affiliations:** 1 Department of Biophysics and Radiobiology, Faculty of Biology, Sofia University “St. Kliment Ohridski”, 8 Dragan Tzankov blvd., 1164 Sofia, Bulgaria; 2 Department of Genetics, Faculty of Biology, Sofia University “St. Kliment Ohridski”, 8 Dragan Tzankov blvd., 1164 Sofia, Bulgaria; 3 Faculty of Mechanical Engineering, Technical University of Sofia, 8 Kl. Ohridski blvd., 1784 Sofia, Bulgaria; 4 Georgi Nadjakov Institute of Solid State Physics, Bulgarian Academy of Sciences, 72 Tsarigradsko Chaussee blvd., 1784 Sofia, Bulgaria

**Keywords:** erythrocytes, pooled immunoglobulin G, hematin, melittin, microelectrophoresis, electrokinetic potential, lipid peroxidation, hemolysis, membrane transport, electrical capacitance, fluorescence microscopy, bilayer lipid membranes

## Abstract

Pooled Immunoglobulin G (IgG), hematin and the membrane-disruptive amphipathic peptide melittin have received attention as powerful biomacromolecules for biomedical and pharmacology applications. Their action on surface properties, oxidation status and epifluorescence properties measured in vitro provide useful information about the functional activity of upper biomacromolecules in erythrocytes in vivo. The hemolysis of erythrocyte membranes, as well as changes in hematocrit and the morphology of erythrocytes, was investigated here via fluorescence microscopy using FITC-concanavalin A binding to cells. The effect of melittin on the membrane capacitance and resistance of model lipid bilayers was probed via electrochemical impedance spectroscopy. Lipid bilayer capacitance was higher in the presence of 0.10 g/L melittin compared to that in the control, which is likely related to bilayer thinning and alterations of the dielectric permittivity of melittin-treated membranes. The biomolecule interactions with red blood cells were probed in physiological media in which the surface of erythrocyte membranes was negatively charged. Surface parameters of erythrocytes are reported upon IgG/hematin and IgG/melittin treatment. Pooled IgG in the presence of melittin, preincubated IgG/hematin preparations promoted a significant decrease in the electrokinetic potential of erythrocytes (Rh-positive). A malondialdehyde (MDA) assay revealed a high rate of lipid peroxidation in erythrocytes treated with IgG/hematin or IgG/melittin preparations. This finding might be a result of pooled IgG interactions with the hematin molecule and the subsequent conformational changes in the protein molecule altering the electrokinetic properties of the erythrocyte membrane related to the Rh group type of erythrocytes. The pooled IgG and hematin are reported to have important consequences for the biophysical understanding of the immunopathological mechanisms of inflammatory, autoimmune and antibody-mediated pathological processes.

## 1. Introduction

Immunoglobulins are widely used in practice [1,2]. There is a tendency to clarify the specific changes of erythrocytes when they are affected by hematin or pooled immunoglobulin G (IgG). The activating or damaging effects of pooled IgG or hematin, as well as the action of melittin as a toxic substance to the erythrocyte membrane, and also the combination of these chemical agents are realized in the erythrocyte through the membrane system of the cells. Pooled IgG is indicated for the treatment of primary humoral immunodeficiencies, such as congenital agammaglobulinemia, common variable immunodeficiency, X-linked agammaglobulinemia, Wiskott–Aldrich syndrome and severe combined immunodeficiencies. Melittin, as the major toxic component of the bee venom from *Apis mellifera*, is used as a membrane-disruptive agent [3] causing injury and hemolysis of the erythrocytes at appropriate concentrations. Melittin is used as a marker for peptide-permeability of erythrocyte membranes. Melittin as an ion-membrane-altering agent is presented, where at certain concentrations, it can act as a tetramer and not just as a monomer when applied at low concentrations affecting the erythrocyte membrane [3,4,5]. Melittin is used as an agent altering the permeable properties of the membrane for ions. Melittin also has hemolytic properties.

The biophysical properties of membranes largely determine the course of physiological and biochemical processes in the cell and are an important link in the morphological changes due to altered properties of erythrocytes in vitro.

Erythrocytes are used as an object to study the properties of biological membranes, as they are easily accessible and have a simple structure. They possess only one membrane and no cell organelles. In the present study, erythrocytes were used as a model system for studying the electrokinetic properties of membranes, and the mechanism of the non-specific binding of polyvalent cations (lectin: FITC-concanavalin A) and pooled immunoglobulin G to the membrane was investigated. A different function of erythrocytes is expected after a change in their morphology after exposure to biomacromolecules. It has been shown that red blood cells undergo morphological transitions from discocytes with thin, doughnut-like shapes to echinocytes and spherocytes using quantitative phase imaging techniques [6]. Melittin induces morphological changes in red blood cells called echinocytosis [6]. Electrical charges on the surface of the cell membrane are an important factor in determining the permeability of the walls of blood vessels, the transport of ions and macromolecules and interactions in intermolecular recognition processes. The surface electrical charge density of membranes and subcellular organelles is decisive in their interaction with the extracellular environment and adhesion and aggregation processes. Electrostatic forces can affect the localization and orientation of integral membrane proteins. Surface charge asymmetry, which arises from ion asymmetry in the bilayer, affects the correct orientation of the protein in the membrane, but transmembrane electric fields affect its conformation. Therefore, the modification of the surface electrical properties through the binding of ions or molecules to the surface of the studied objects in many cases leads to a number of changes in the molecular organization of the membrane and its biochemical and biophysical properties [7].

Therefore, the study and analysis of the electrokinetic characteristics of membranes and the possibility of influencing them are important in optimizing the conditions of interactions of membranes with other molecules and aggregation in some cells.

The biophysical state of the erythrocyte membrane is directly related to the surface charge, the presence of which can be calculated based on the electrophoretic mobility and zeta potential of the cell [8,9]. The registration of the movement of blood cells in a constant electric field under physiological conditions of the suspension medium allows an evaluation of the functions of the erythrocyte membrane. A pronounced change in the electrophoretic mobility of erythrocytes is found in normal and pathological conditions [10,11]. The electrokinetic potential of cells is genetically determined, and its decrease is associated with the aggregation of erythrocyte membranes. A number of pathological processes are associated with a modification of the electrophoretic mobility and zeta potential of erythrocytes. Electrophoretic mobility is a measure of the magnitude of the surface electrical charge of cells without or in the presence of physical factors or chemical agents. General biophysical regularities at the membrane level may cause changes in the electrophoretic mobility, zeta potential and surface electrical charge of erythrocytes upon exposure to pooled IgG and hematin, which needs more studies. When various chemical agents are applied to the erythrocyte membranes, which are released during inflammation and/or in the presence of pooled immunoglobulin G, as well as the membrane-disruptive melittin in the presence of IgG, molecular changes to the plasma membrane of the cell are observed. Erythrocyte membranes are the immediate target of the damaging action of a number of factors in the pathological process of the erythrocyte life cycle together with the initiation of lipid peroxidation as a universal mechanism of cell damage [9,12]. The application of additional biophysical studies on erythrocyte membranes after exposure to pooled IgG, hematin and melittin could be a contribution to clarifying the mechanism of membrane alteration. The physical parameters of the interaction of melittin with model membranes need to be investigated via electrochemical impedance spectroscopy [13].

Complex analysis of the electrokinetic properties of human erythrocytes in terms of dependence on Rhesus factor and other biophysical characteristics will allow us to obtain a general picture of the main mechanisms of action of hematin and pooled IgG on the functional activity of erythrocytes and the causes of the damaging effect of these biomacromolecules, of which actions on biological membranes have not been explained in detail. Rhesus (Rh) factor is a clinically significant blood group system in transfusion medicine [14]. With different alterations to pooled IgG and hematin, as well as combinations of these agents, the action of melittin in the presence of pooled IgG on the erythrocyte membrane, evidenced by changes in the morphology of erythrocytes with biomacromolecules, hemolysis and acid-induced stability of erythrocytes, hematocrit and lipid peroxidation in the presence of these biological active agents is expected.

The disclosure of the biophysical mechanisms of changes in the erythrocyte membrane is extremely important in order to assess the state of interactions of hematin, pooled IgG and hematin, as well as pooled IgG and melittin, to evaluate the functional state of the cell and to develop methods for diagnosing the inflammatory process.

Based on the above, the aim of the present work was to study the electrokinetic properties of human erythrocytes and their interrelationship with the structural–functional state of the membrane in the norm and after influencing the functions with the biological active macromolecules pooled immunoglobulin G, hematin and melittin in vitro.

The present study will focus on the possibility of proving the use of electrokinetic parameters as a method for diagnosing inflammatory reactions in the body, related to the release of hematin and its interaction with pooled IgG in response to the applied impact (physical factors and chemical agents). The significance of biomacromolecule interactions with erythrocyte membranes provides a potential explanation of inflammatory diseases and anti-oxidative stress. The presented work would have future fundamental applications in biomedicine.

## 2. Materials and Methods

### 2.1. Materials

All chemicals used in the present study were of analytical grade. OCTAGAM^®^ IVIG 5%, 2.5 gm in a 50 mL vial, (Human) Immune Globulin Intravenous—5% solution liquid preparation—was purchased from Octapharma International Services N.V., Octapharma N.V., De Frélaan 269/4, B-1180 Brussel. Human Immunoglobuline (50 g/L; min 95% IgG) 2.5 g/50 mL—Maltose 5 g—water injection contained the following subfractions: IgG_1_ 62.6%, IgG_2_ 30.1%, IgG_3_ 6.1%, IgG_4_ 1.2%. Melittin from bee venom, 65–85% (HPLC); FITC-CoA, concanavalin A FITC-labeled from *Canavalia ensiformis* (Jack bean) Type IV; Na_2_HPO_4_, sodium phosphate dibasic; KH_2_PO_4_, potassium phosphate monobasic were purchased from Sigma-Aldrich (St. Louis, MO, USA), and the chemicals were as follows: NaCl, KCl, NaOH; TCA (trichloroacetic acid); 2-thiobarbituric acid (TBA); NaN_3_, sodium azide; DMSO, dimethyl sulfoxide (methylsulfinylmethane); Hematin—Koch-Light Laboratories Ltd., Colnbrook, Bucks, UK. Propidium iodide was purchased from VECTOR Laboratories Inc. 6737 Mowry Ave Newark, CA 94560, USA. D (+)-Saccharose was purchased from Riedel-De Haën AG, Seelze, Hannover, Germany. Egg-yolk L-α-phosphatidylcholine (egg PC, 840051P) was from Avanti Polar Lipids Inc. Alabaster, AL, USA). Pentane CH_3_(CH_2_)_3_CH_3_ and hexane CH_3_(CH_2_)_4_CH_3_ of HPLC grade were supplied from Honeywell, Riedel-de Haën AG, Seelze, Hannover, Germany. Bidistilled water from a quartz distiller for the preparation of all aqueous solutions was used. All buffer media solutions were previously filtered through Whatman^®^ membrane filters PTFE, cellulose nitrate, pore size 0.2 µm, diam. 47 mm, non-sterile, Whatman 7402-004, Whatman Article № 28420767, Sigma Aldrich, Merck KGaA, Darmstadt, Germany.

A hematin (ferriprotoporphyrine IX) stock solution was prepared in DMSO to a final concentration of 2.5 mM [15]. Pooled immunoglobulin G preparations were diluted in PBS to 10 mg/mL [16]. All treated preparations were stored at 4 °C in the presence of 0.1% NaN_3_.

### 2.2. Isolation of Erythrocytes

Erythrocytes collected from complete blood samples taken from healthy adult subjects were supplied by the National Centre of Hematology and Perfusion, Sofia, Bulgaria. Venom blood samples were taken from healthy human subjects, using EDTA-anticoagulant vacutainers for the study. The present study complied with the ethical regulations and legislation in both Europe and Bulgaria. The experiments were performed in compliance with “WMA Declaration of Helsinki, Ethical Principles for Medical Research Involving Human Subjects” [17]. All subjects recruited for the study provided written informed consent. Ethic Committee Name: Ethics Committee of Sofia University “St. Kliment Ohridski”, Approval Code:RD-04-91, date 25 February 2022.

Erythrocytes were centrifuged in a microcentrifuge Eppendorf^®^ MiniSpin^®^ with standard Rotor, Eppendorf AG, Hamburg, Germany at 2500 rpm for 5 min and were washed 3 times with phosphate-buffered saline (10.1 mM Na_2_HPO_4_, 1.8 mM KH_2_PO_4_, 136.9 mM NaCl, pH 7.4). Erythrocytes (hematocrit 0.20) were used within 4 h after preparation. We used phosphate-buffered saline (PBS, pH 7.4), containing 137 mM NaCl, 10.1 mM Na_2_HPO_4_ and 1.8 mM KH_2_PO_4_ for the determination of hematocrit, hemolysis (Hb-release) and HCl-hemolysis, membrane transport, electrokinetic parameters, lipid peroxidation and fluorescence microscopy measurements, respectively, where the stock suspension of erythrocytes was then diluted to 1% hematocrit. Erythrocytes were treated with biomacromolecules for 1 h at 37 °C, at a mixing speed of 300 rpm (TMix Thermalmixer, Analytic Jena AG, Jena, Germany). The effect of fixed concentrations of pooled immunoglobulin G (10 mg/mL), hematin (80 µM [15]) and melittin (0.2 µg/mL Mt; 0.5 µg/mL Mt; 2 µg/mL Mt and 5 µg/mL Mt) treatments on biophysical parameters (average value ± SD) of erythrocytes with a Rhesus-positive factor and Rhesus-negative factor were investigated.

### 2.3. Hematocrit and Hemolysis Tests

We determined hematocrit via microcentrifugation (NF 048 NÜVE SANAYİ MALZEMELERİ İMALAT VE TİCARET A.Ş bench-top centrifuge, Akyurt—Ankara, Türkiye. Hematocrit adjustment was performed via blood centrifugation (12,500× *g* for 2 min), plasma removal and addition of cells in the desired ratio to obtain 20% (*v*/*v*). The samples were aliquoted into Eppendorf tubes and homogenized. Tubes (Heparin Disposable Pipettes, containing 50 microliters, 67 mm length, made in USA, by Drumond Scientific Company, distributed by Rudolph GRAVE AB Stockholm, Sweden) of precise bore glass were filled with the human erythrocyte suspension, one end was sealed and the tubes were centrifuged for the constant packing of cells [9]. The relative heights of the packed cell column and the total fluid were measured using a special optical device.

The hemolysis test at different concentrations of NaCl to determine the degree of hemolysis was used. The erythrocyte hemolysis test was fixed [18] by adding erythrocytes to a series of hypotonic solutions with decreasing NaCl concentrations (0.9–0.3% NaCl) at 5% Hct, with incubation for 30 min at 25 °C with gentle mixing (speed of 300 rpm using TMix, Analytic Jena AG, Jena, Germany). Afterward, 1.5 mL of the samples with different salt concentrations were diluted with the samples of the erythrocyte suspensions previously incubated with biomacromolecus (1 h at 37 °C) at 25 °C for 30 min. The erythrocytes incubated with different hypotonic solutions were then centrifuged (12,000× *g* for 1 min), and after that, the supernatants were removed. The hemoglobin of each supernatant was measured from the absorbance at λ = 576 nm immediately after centrifugation [19] using a BOECO Spectrophotometer S-200 (VIS), Boeckel + Co (GmbH + Co) KG, Hamburg, Germany. The Abs (absorbance) was calculated by plotting the relationship between the absorbance at 576 nm, respectively, versus the absorbance at the appropriate concentration of the NaCl solution.

The value of ΔHemolysis  was calculated as follows:

(1) $$\Delta Hemolysis=\frac{\left(Abs_{biomacromolecules,x}-Abs_{biomacromolecules,0.9\%\;NaCl}\right)}{Abs_{biomacromolecules,x}}$$

where $Abs_{biomacromolecules,x\;}$ is the absorbance Abs _(λ=576)_ of the release of hemoglobin from erythrocytes without or in the presence of different concentrations of biomacromolecules, measured at λ = 576 nm and $Abs_{biomacromolecules,0.9\%\;NaCl}$ is the absorbance from the release of hemoglobin in erythrocytes without or in the presence of biomacromolecules, suspended into 0.9% NaCl solution, respectively.

### 2.4. Acid—Hemolytic Stability Assay

The acid-induced resistance of human erythrocytes has been studied in isosmotic media (8.5% NaCl) in the presence of 0.004 N HCl as a hemolytic agent. Erythrograms were obtained according to the classical method [20]. The method of erythrograms is described in detail. In a blood sample obtained by diluting the blood with physiological solution about 10^3^ fold, an acid hemolytic is placed, of which the chemical composition and concentration can be determined very precisely. Hydrochloric acid is used as this hemolytic. From the moment the hemolytic is introduced, the transparency of the sample is periodically measured, which gradually increases and after some time reaches a relatively high and constant value. The dependence of transparency on time is an integral curve that reflects the transition from the initial transparency of the sample to the final one. According to Terskov and Gitelzon [20,21], this integral curve is not sufficiently representative of the hemolytic process, and therefore, the authors replaced it with the first and time derivative, which expresses the dependence of the instantaneous rate of hemolysis on time. Since the measurements of the transparency of the sample during the hemolytic process are made only at individual moments, both the integral and the derivative curve have a polygonal character. The derived curve is called the acid erythrogram [21]. The study of the hemolytic process is conveniently performed spectrophotometrically. The duration of the hemolytic process depends on the properties of the erythrocyte membranes, on the type and concentration of the hemolytic and on the temperature of the sample during hemolysis.

The difference between the absorbance of the erythrocyte membranes at the beginning of the process of hemolysis and the final value of absorbance represents the change in the absorbance coefficient, and we approximated its value as 100 per cent. The changes between two following absorbance values are represented as the percent of the whole change in absorbance. The upper values are proportional to the average velocity of the hemolytic process during every separate interval of 30 s between the 2 following measurements. The graph dependence of the changes in acid-resistance (partial hemolysis, hemolyzed erythrocyte fraction (%)) in time during 10 min represents the acid-induced erythrogram. Consequently, we used the acid erythrogram representing the 1st derivative of the optical density kinetic curve and characteristics of the erythrocyte population heterogeneity as in [20,22].

### 2.5. Microelectrophoresis

The electrophoretic mobility (EPM) measurements were performed using the microelectrophoresis technique with the OPTON Cytopherometer (Feintechnik Ges, m.b.H., Zeiss-Opton, Oberkochen, Germany). Electrophoretic mobility was measured in a rectangular chamber and platinum electrodes at a constant electric field of 5 mA and temperature of 25 ± 0.1 °C. The movement of erythrocytes over a known distance (16 μm) was timed for both forward and backward (reversed field) runs. The erythrocytes were observed under a light microscope connected to a Sony video camera (Video Camera Head CH–1400 CE, Sony Corporation, Japan) providing 2000 times magnification and a JVC monitor (Victor Company of Japan, LTD., Yokohama, Japan). The results were expressed by means of the EPM (per 10^−8^ m^2^ V^−1^s^−1^) ± standard deviations (SD) for each sample. The electrical conductivity and viscosity of the different erythrocyte suspensions were measured using a Thermo Fisher Scientific CyberScan PC 510 (Oakton^®^ Instruments/Eutech Instruments Pte Ltd., Singapore) pH/Conductivity meter and a Rheo (VEB MLW Prüfgeräte–WERK, MEDINGEN/SITZ FREITAL/GDR, Typ 202, Germany) viscometer, respectively. Values represent the mean of three replications (54–96 erythrocytes). The electrokinetic (zeta) potential (ζ) was calculated from the electrophoretic mobility, *u*, using the Helmholtz–Smoluchowski equation [23]:

(2) $$\zeta =\frac{\eta u}{\varepsilon_{r}\varepsilon_{0}}$$

where ζ is in units of mV, $\varepsilon_{r}=78.5\;\left(\mathrm{at}\;25\;{}^{\circ}C\right)$ is the relative dielectric permittivity of the aqueous phase, $\varepsilon_{0}=8.8542\times 10^{-12}Fm^{-1}$ is the permittivity of free space and η=0.001 Pa.s is the viscosity of the PBS: 136.9 mM NaCl, 10.1 mM Na_2_HPO_4_, 1.8 mM KH_2_PO_4_, pH 7.4 (pH 7.4 at 25 °C) as in [24].

The electrostatic potential in the aqueous phase of the erythrocyte membrane surface and charge density (*σ*) is given by the following:

(3) $$\frac{A\sigma }{\surd C}=sinh\left(\frac{ze\Psi o}{2kT}\right)$$

where Ψo  is in mV, A=136.6 at 25 °C, $A=1/\surd \left(8N\varepsilon_{r}\varepsilon_{0}kT\right)$, N=6.022×10^23^ mol^−1^ is the Avogadro constant). The surface electrical charge of erythrocytes is expressed in C/m^2^. The values of surface charge density were calculated according to the assumption that ζ≅Ψo [24]. The electrophoretic mobility of the cells gives information about the dynamics of the surface electrical charge on the outer membrane surface, i.e., the average amount of electrical charges that is generated on the outer surface of the cell membrane of the erythrocyte was calculated. The surface electrical charge was calculated from the zeta potential of the cell by making the approximation that the electrokinetic potential is approximately equal to the surface or electrostatic potential of the cell according to the Gouy–Chapman theory. This explicitly states that the value of the zeta potential depends on the ionic strength. Therefore, erythrocytes were placed in a medium with an isotonic ionic strength to follow the change in the electrokinetic parameters of the erythrocyte membrane in the presence of pooled immunoglobulin G, hematin and a combination of pooled immunoglobulin G and melittin. The Gouy–Chapman theory was used in the calculation of the surface charge used in the determination of the surface electrical charge. The surface electrical charge of erythrocytes was determined to obtain a complete evaluation of the change in surface electrostatics without and in the presence of biomacromolecules in the suspending medium.

### 2.6. Measurements of Proton Transport

The maintenance of cellular homeostasis in the conditions of great differences in the chemical composition of the cytoplasm and the environment is ensured by the barrier functions of the membrane and the selective transport of substances in the cell. Erythrocytes can be considered systems in a non-equilibrium steady state. Under normal conditions, anion transport across the membrane is maintained in a certain equilibrium state. When the erythrocytes are transferred from saline to an isotonic sucrose solution of low ionic strength, a spike in the curve is observed at the beginning of the experiment, which is due to the escape of the intracellular solution. To some extent, of course, there is an antiport of chloride and bicarbonate ions dissolved in the medium (the suspension is in continuous contact with air). However, the time constants of this process are too large. In unbuffered solutions, the adaptive process is too fast and the new C-state is reached in less than 2 min. The equilibrium of the cells is mainly determined by the change in the external pH. This process can be explained, at least in part, by the minimal amount of OH^−^ and H^+^ ions that must be transported from inside to outside the cell to establish equilibrium. The following processes are possible: (1) OH^−^/Cl^−^ antiport or H^+^/Cl^−^ cotransport; (2) Cl^−^ antiport with divalent ions. The transfer of erythrocytes into solutions with altered electrolyte content induces passive ion transport processes. According to Glaser and coworker’s model [25] of ionic states in human erythrocytes, a quasi-equilibrium “C-state” exists, characterized by an equilibrium of all permeant anions and protons, but maintaining the initial Na^+^ and K^+^ contents. The kinetics of reaching this C-state is determined based on anion fluxes and components of the pH-equilibrium and is therefore limited by membrane permeability to them, as well as driving forces. In the presence of CO_2_, HCO_3_^−^ and carboanhydrase, the equilibrium for Cl^−^ ions and pH is mediated by the highly efficient Jacobs–Stewart cycle. In most cases, the concentration of carboanhydrase, CO_2_ and HCO_3_^−^ in the experimental solutions is much lower than that under physiological conditions. Therefore, under in vitro conditions, the unimpaired transport of Cl^−^ ions via the Jacobs–Stewart cycle cannot be expected, and the electroneutrality of the equilibrium process can only be maintained via the co-transport of Cl^−^ and H^+^ or via their antiport against other anions OH^−^.

The experimental study on the anion–proton co-transport was based on the measurement of net proton flows associated with erythrocyte Band 3-mediated net anion transfer. An erythrocyte suspension (500 µL, Hct = 20%) of previously incubated erythrocyte membranes (without or in the presence of biomacromolecules at 37 °C for an hour incubation and preincubation of pooled immunoglobulin G (10 mg/mL) and 80 µM hematin) was added to 50 mL of unbuffered isotonic sucrose medium, and the time course of the pH of the medium was registered for 10 min. The changes in pH are presented as a percent of the control. All experiments were carried out at 25 °C.

Erythrocytes suspended in hypotonic sucrose solution can be characterized by the exchange of inorganic anions of chloride and carbonate in connection with the pH equilibration, which occurs in a minutes. Water transport in an erythrocyte suspension equilibrates the osmotic gradient in less than one second [25]. Erythrocytes are characterized by a “C-state” with a stable quasi-stationary state for hours. We measured the extracellular proton concentration (H^+^_ext_) as a function of time in seconds promoted by the treatment of erythrocytes with biomacromolecules as in [26]. The suspending medium of 0.3 M sucrose (NaOH), pH 7.4, to maintain its buffer capacity constant over the pH range covered in the erythrocyte experiments was used [25].

Proton efflux began by mixing 500 µL of the erythrocyte suspended into 50 mL of 0.3 M sucrose (NaOH), pH 7.4. The pH of the erythrocyte suspension without or in the presence of different concentrations of biomacromolecules at the same time was measured using a Thermo Fisher Scientific CyberScan PC 510 (Oakton^®^ Instruments/Eutech Instruments Pte Ltd., Singapore) pH/conductivity meter.

The results of the proton efflux alterations in extracellular media in the presence of different concentrations of biomacromolecules were obtained from membrane transport measurements in erythrocyte suspending media every 20 s during 10 min with gentle mixing. The value of ΔpH(%) was calculated from the following:

(4) $$\Delta pH=\frac{\left(pH_{o}-pH_{t}\right)}{pH_{0}}\times 100\;\;\;\;\;$$

where $pH_{o}$ and $pH_{t}$ are the pH values of erythrocytes in biomacromolecule-free medium and in the presence of biomacromolecule concentrations, respectively.

### 2.7. Lipid Peroxidation Determined with Thiobarbituric Acid Reactive Substances (TBARS)

Malondialdehyde content was measured according to [27,28] with modifications. The erythrocyte suspensions (500 μL erythrocytes in PBS, pH 7.4, 2 mM NaN_3_, Hct = 20%) without or in the presence of biomacromolecules after an incubation and preincubation time period were homogenized in 400 μL of 28% trichloroacetic acid (TCA) and centrifuged at 12,500 × *g* for 4 min. After centrifugation, 1 mL of supernatant was mixed with 500 μL of 1% thiobarbituric acid (TBA) in 1% NaOH, and the mixture was incubated in boiling water for 30 min. The suspending medium was centrifuged at 12,500× *g* for 2 min, and the absorbance of λ = 532 nm was measured using a BOECO Spectrophotometer S-200 (VIS), Boeckel + Co (GmbH + Co) KG, Hamburg, (Germany) to determine the MDA content. The thiobarbituric acid-reactive substance (TBARS) molar concentration, *c*, was calculated as follows:

(5) $$c=\frac{Abs}{\varepsilon l}\;\;\;\;\;\;\;\;\;\;$$

where Abs is the absorbance, ε stands for the molar absorption coefficient of H_2_O_2_, $\varepsilon_{532}=154,000\;M^{-1}{\mathrm{cm}}^{-1}$ and l represents the optical path length. Afterwards, the reaction was stopped by cooling the samples in an ice bath. MDA reacts in the TBA test to generate a colored product. In acid solution, the product absorbs light at 532 nm. MDA content was estimated by using an extinction coefficient of 154 mmol L^−1^ cm^−1^. The lipid peroxidation of erythrocyte membranes was determined based on the production of TBARS and expressed in μmol L^−1^ [27].

### 2.8. Fluorescence Microscopy Studies

The samples were observed using a Zeiss Axioscope 5 microscope with a fluorescence LED Illumination Colibri 3 (Carl Zeiss Microscopy GmbH, Jena, Germany) at 1000× magnification (objectives 100× and eyepieces 10×) under immersion, with appropriate excitation filters for propidium iodide (PI) staining solution and fluorescein isothiocyanate (FITC): blue, 478–495 nm. The results were documented with an Axiocam 202 mono digital camera (Carl Zeiss Microscopy GmbH, Jena, Germany) and ZEN 2.5 (blue edition) software (Carl Zeiss Microscopy GmbH, Jena, Germany). Fluorescein isothiocyanate (FITC)-labeled lectin concanavalin A (CoA) was used. Concanavalin A-FITC labeled from *Canavalia ensiformis* (Jack bean) Type IV, FITC content of 3.6 mol/mol lectin (Mol.Wt of lectin approx. 102,000) has an affinity for terminal α-D-mannosyl and α-D-glycosyl residues. ConA-FITC inhibitory carbohydrates are α-methylmannoside and α-methylglucoside. FITC-Con A (MW 102,000 Da) labeling solutions were dissolved in distilled water at 2 mg/mL as a stock solution in dark tubes. They were diluted with PBS, pH 7.4, to 40 µg/mL before use. Erythrocyte membranes were transferred into the PI staining solution. After 60 min of incubation at 37 °C, erythrocytes (2 × 10^6^ cells/mL) were washed with PBS (15 min 12,000× *g*, Micro22R, Andreas Hettich GmbH & Co. KG, Tuttlingen, Germany)) and examined under via epifluorescence using a Zeiss Axioscope 5 (Carl Zeiss Microscopy GmbH, Jena, Germany).

Fluorescence microscopy of erythrocytes with FITC-labeled CoA samples followed the protocol: microscopic preparations made of all samples in duplicate, 20 μL of each sample, in separate Eppendorf tubes, were placed in the resuspended sediment with erythrocytes in 100 μL PBS, pH 7.4. Then, 15 μL of propidium iodide solution (PI, 1.0 mg/mL solution in water, Invitrogene, Thermo Fisher Scientific Inc., Eugene, OR, USA, cat. P3566) was added to each. Samples were incubated for 30 min at 37 °C and then centrifuged at 14,000 rpm for 5 min. The pellet of each sample was resuspended in 35 μL of PBS buffer, and the entire amount was spotted onto a glass slide coated with poly-L-lysine (poly-L-lysine is coated glass slides, Poly-Prep Slides, Sigma-Aldrich, Merck, Saint Louis, MO, USA, cat. P0425-72EA) and covered with cover glass. The preparations were placed in a humid chamber (a box at the bottom of which there were several layers of filter paper wetted with PBS), wrapped with aluminum foil. The preparations were incubated at +4 °C, overnight. For better stabilization of the fluorescent signal in erythrocytes without and in the presence of biomacromolecules, they were allowed to stain better for the formation of a luminescent halo around the erythrocyte, which does not form immediately after incubation with FITC-concanavalin A. The cells were faintly stained, and their luminescence was not immediately noticeable after treating them with FITC-labeled lectin on the same day after incubation following the protocol for labeling them. Letting the treated samples stand with FITC-concanavalin A was necessary for better fluorescence microscopy images and better light intensity, where no change in the morphology of the cells was observed after they were stained in the solution overnight. The results were documented with an Axiocam 202 mono digital camera (Carl Zeiss Microscopy GmbH, Jena, Germany) and ZEN 2.5 (blue edition) software (Carl Zeiss Microscopy GmbH, Jena, Germany) [29].

### 2.9. Electrochemical Impedance Spectroscopy of Bilayer Lipid Membranes

We studied the effect of melittin on the electrical properties of bilayer lipid membranes (BLM) formed via the Montal–Mueller method [30,31] from egg-yolk L-α-phosphatidylcholine. Pentane CH_3_(CH_2_)_3_CH_3_ and hexane CH_3_(CH_2_)_4_CH_3_ of HPLC grade were used for dissolving the lipid. The Montal–Mueller chamber, model BC-20A, was provided by Eastern Scientific LLC (Rockville, MD, USA). Solvent-free bilayers were obtained suspended on the aperture with a diameter of 100 µm in a 0.025 mm thin Teflon membrane according to the corresponding protocol [30] as described in detail in [31]. The final control of the bilayer formation and its quality were verified electrically [32]. This was proceeded to the fast Fourier transform electrochemical impedance spectroscopy (FFT-EIS) measurement immediately afterwards. A multisine perturbation signal was applied, characterized by a small amplitude ~10 mV in the frequency range of 1.5 Hz–50 kHz. The measurement procedure comprised the simultaneous acquisition of the perturbation and the respective response signals followed by their fast Fourier transformation (FFT) to the frequency domain. This approach allowed the impedance spectrum to be acquired in a couple of seconds and the stationarity to be simultaneously monitored [33,34], which is particularly appropriate for probing the impedance characteristics of lipid membranes.

The capacitance $C_{\mathrm{BLM}}$ and resistance $R_{\mathrm{BLM}}$ of the planar lipid membrane were deduced from the analysis of the acquired FFT-EIS data taking into account the equivalent circuit representing the Montal–Mueller BLM configuration as discussed in [31]. The membrane specific capacitance $C_{m}=C_{\mathrm{BLM}}/S_{m}$ and resistance $R_{m}=R_{\mathrm{BLM}}S_{m}$ were calculated from the measured capacitance and resistance of the bilayer and its 100 µm-circle surface area $S_{m}=$ 8 × 10^−5^ cm^2^. The values reported below were calculated as the weighted average of at least 3 independent measurements averaged over 10 repetitions each.

### 2.10. Statistical Analysis

The electrokinetic data were averaged from triplicate measurements for every sample. The data are expressed as the mean ± SD. The significant means were determined by use of ANOVA. One-way analysis of variance was performed with Dunn’s Test following ranked-based ANOVA (Kruskal–Wallis One Way Analysis of Variance on Ranks) and the Student–Newman–Keuls method taking *p* < 0.05 as significant, *p* < 0.01 as highly significant and *p* < 0.001 as extremely significant and represented by an asterisk in the figures. Statistical analyses were also performed using Minitab v.17.

## 3. Results

### 3.1. Hematocrit and Hemolysis Tests

#### 3.1.1. Hematocrit

**Figure 1:**
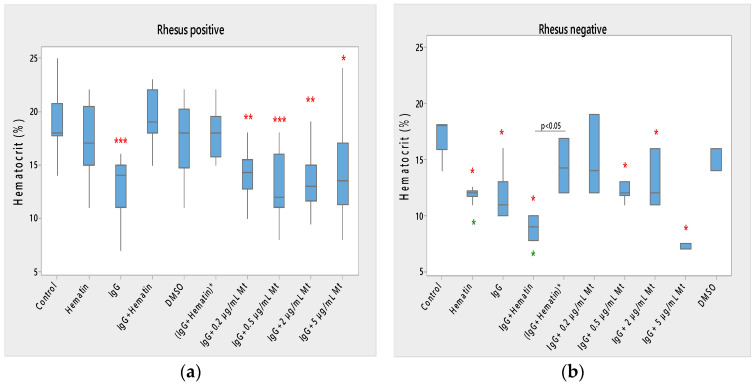
Effect of fixed concentrations of pooled immunoglobulin G (10 mg/mL), hematin (80 µM) and melittin (0.2 µg/mL Mt; 0.5 µg/mL Mt; 2 µg/mL Mt and 5 µg/mL Mt) treatments on hematocrit (average value ± SD) of erythrocytes (**a**) with an Rhesus-positive factor and (**b**) Rhesus-negative factor. The pre-incubated sample of pooled immunoglobulin G (10 mg/mL) with hematin (80 µM) is indicated by an asterisk on the abscissa. Erythrocytes were treated with biomacromolecules for 1 h at 37 °C in phosphate buffered saline (PBS), pH 7.4. Hematocrit determination was performed at 25 °C. Each value is the mean ± SD of three independent preparations with 8–15 repetitions each. *** *p* < 0.001; ** *p* < 0.01; * *p* < 0.05 compared to the untreated control (red asterisks) or DMSO (80 µM)—control (green asterisks).

Hematin did not significantly affect the hematocrit of Rh-positive erythrocytes but strongly decreased the hematocrit in Rh-negative erythrocytes (Figure 1) compared to control values without hematin and compared to control values in the presence of DMSO (* *p* < 0.05). A comparison of the effect of dimethyl sulfoxide on the surface properties of human erythrocytes is needed, as it may affect the electrokinetic parameters of the erythrocyte membrane. At the same time, it is necessary to compare the effect of hematin, pooled immunoglobulin G and hematin, as well as their preincubation, on the surface characteristics of the membrane, when there should be a second control of dimethyl sulfoxide in which the hematin is dissolved. This second control is necessary to accurately describe the action of hematin, pooled immunoglobulin G and hematin, on the electrokinetic parameters, where the action of dimethyl sulfoxide in the second control variant is accounted for. The data thus obtained enrich the information about the surface properties of erythrocytes under the influence of biomacromolecules.

Pooled immunoglobulin G (10 mg/mL) significantly reduced the hematocrit of Rh-positive erythrocytes (*** *p* < 0.001). Pooled IgG significantly decreased the hematocrit of Rh-negative erythrocytes (* *p* < 0.05) compared to control values, and the observed change did not differ from the effect of hematin on the volume percentage of the red blood cells in the blood (Figure 1a,b).

The combination (IgG+hematin) did not affect the hematocrit of erythrocytes (Rh-positive). The (IgG+hematin) preparation caused a decrease in the hematocrit of erythrocytes with the Rh-negative factor to 8.80 ± 0.97% (* *p* < 0.05) compared to control values without hematin and compared to control values in the presence of DMSO (* *p* < 0.05) (Figure 1b).

No changes are found in the hematocrit of erythrocytes with the Rh-positive factor after exposure to DMSO.

“IgG + 0.2 µg/mL Mt” treatment of Rh-positive erythrocytes was characterized by decreased hematocrit compared to control values (** *p* = 0.004), and this decrease was similar to the decrease in erythrocyte hematocrit after pooled IgG treatment and was due to its protective action.

In studies with biomacromolecules, it was established that pooled IgG caused a strong decrease (*** *p* < 0.001) in the hematocrit of the erythrocyte suspension with a Rhesus-positive factor, and additional treatment with different concentrations of melittin also led to a decrease in hematocrit (Figure 1a).

It was found that pooled IgG (10 mg/mL) in the presence of 0.2 µg/mL melittin reduced the hematocrit value of erythrocytes with the Rh-positive factor (** *p* = 0.004). The sample containing pooled IgG and 0.5 µg/mL melittin significantly lowered the hematocrit of the erythrocyte suspension (*** *p* < 0.001) compared to the control value without chemical agents. Higher concentrations of melittin treatment on erythrocyte suspensions containing pooled IgG also decreased hematocrit compared to control values, as in the case of pooled IgG and 2 µg/mL melittin (** *p* = 0.003), and pooled IgG and 5 µg/mL melittin reduction this parameter (* *p* = 0.016).

Therefore, a decrease in the hematocrit of Rh-positive erythrocytes was observed in the presence of the pooled IgG and treatment with all tested concentrations of melittin in the suspension medium (Figure 1a).

#### 3.1.2. Hemolysis of Erythrocytes in the Presence of Biomacromolecules

Data about ∆Hemolysis (Hb-release) under biomacromolecule treatment were characterized by 30 min time periods of incubation in different concentrations of salt media according to the protocols described after the incubation of erythrocytes and biomacromolecules for 1 h at 37 °C, as well as after the preincubation of pooled immunoglobulin G and hematin in PBS, pH 7.4, for 1 h at 37 °C after gentle mixing. According to our results and the data of [35], no blood hemolysis was expected and observed up to one week after its storage before our measurements.

Figure S1a,b represents the results for the hemoglobin released (Hb-release) (∆Hemolysis, r.u.) in the presence of fixed concentrations of pooled IgG, hematin and melittin and the variants, including a combination of chemical compounds, noticed as *biomacromoleculs,* respectively, as obtained from hemoglobin release at λ = 576 nm in suspending media with different NaCl concentrations.

##### Hemolysis of Erythrocytes with the Rh-Positive Factor

Erythrocytes that had not been treated with biomacromolecules, called controls, were characterized by a sharp decrease in changes in hemoglobin release in low concentrations of NaCl (0.3%) medium and showed insignificant changes in the hemolysis of the cells until reaching changes of ∆H = 10.06 in 0.3% NaCl (Figure S1a).

Rhesus factor-positive hematin-treated erythrocytes had weak changes in hemoglobin release in environments of 0.9–0.4% NaCl, reaching a change of ΔH = 7.68 at 0.3% NaCl.

Pooled IgG did not cause significant changes in the release of hemoglobin from erythrocytes with a Rhesus-positive factor, which were suspended in media of 0.9–0.4% NaCl. There was a large change in the hemolysis of Rh-positive erythrocytes in the presence of 10 mg/mL IgG (∆H = 52.18) compared to the release of hemoglobin from erythrocytes in 0.9% NaCl.

The changes in the hemolysis of erythrocytes with a Rhesus-positive factor in the presence of pooled IgG and hematin, as well as after exposure to pre-incubated pooled IgG with hematin, were slightly expressed. Hemolysis values were ∆H = 1.5 (IgG+hematin) and ∆H = 1.2 (IgG+hematin)*.

DMSO did not cause changes in the hemolysis of erythrocytes with the Rh-positive factor in the concentration range of 0.9–0.45% NaCl. Strong changes were observed in the release of hemoglobin from Rh-positive erythrocytes after treatment with DMSO in 0.4% NaCl (∆H = 24.07) and 0.3% NaCl (∆H = 9.78) compared to the value of hemolysis from erythrocytes in 0.9% NaCl.

The “IgG + 0.2 µg/mL melittin” preparation caused a strong alteration in the release of hemoglobin from erythrocytes with a Rhesus-positive factor, where they reached changes in hemolysis from ∆H = 27.96 in 0.3% NaCl to ∆H = 5.41 in 0.4% NaCl.

The “IgG + 0.5 µg/mL melittin” preparation resulted in 3.9% hemoglobin release from Rh-positive erythrocytes suspended in 0.4% NaCl. “IgG and 0.2 µg/mL melittin” produced a 5.4% hemoglobin release from Rh-positive erythrocytes in 0.4% NaCl and a 3.7% increase in the ∆H of erythrocytes in 0.55% NaCl. “IgG and 0.5 µg/mL melittin” caused the release of hemoglobin from erythrocytes in 0.60% NaCl.

“IgG and 2 µg/mL melittin” and “Ig and 5 µg/mL melittin” suppressed the Hb release from erythrocytes with a Rhesus-positive factor in almost all solutions with tested concentrations of NaCl (0.4–0.9%).

##### Hemolysis of Erythrocytes with the Rh-Negative Factor

Erythrocytes with a Rhesus-negative factor without biomacromolecules placed in a medium containing 0.9% NaCl–0.3% NaCl were characterized by the following features: 0.8% NaCl led to an increase in the release of hemoglobin (∆H = 6.78) (Figure S1b). Hemolysis at 0.45% NaCl was ∆H = 5.53 and ∆H = 8.37 in 0.4% NaCl. The strongest was the change in the release of hemoglobin from erythrocytes with a Rhesus-negative factor in 0.3% NaCl, where ∆H = 23.61 compared to the hemolysis of cells in 0.9% NaCl.

Hematin did not cause changes in the hemolysis of erythrocytes with the Rh-negative factor in a medium containing 0.9–0.45% NaCl. The change in ΔH in the presence of 0.4% NaCl was insignificant, but at 0.3% NaCl, it reached a change in the release of hemoglobin from the cells (∆H = 11.29).

Pooled IgG caused weak changes in the release of hemoglobin from cells in a medium containing 0.9% NaCl–0.45% NaCl. At 0.5% NaCl, a slight increase in the release of hemoglobin from erythrocytes with a Rhesus-negative factor was observed (∆H = 5.43). A strong increase in hemoglobin release from cells treated with pooled IgG (10 mg/mL) was recorded in 0.3% NaCl. A similar dependence was observed in Rh-negative erythrocytes after treatment with pooled IgG and hematin. There was a slight change in hemolysis (∆H = 5.19) in 0.4% NaCl and a significant increase in the release of hemoglobin from erythrocytes with the Rh-negative factor in 0.3% NaCl (∆H = 26.09).

Preincubated pooled IgG with hematin resulted in a strong reduction in hemolysis (∆H = 9.16) in contrast to that in the sample with pooled IgG and hematin and no changes in saline medium of 0.9–0.4% NaCl.

Rhesus-negative erythrocytes treated with DMSO were characterized by a strong increase in hemoglobin release from cells in 0.3% NaCl (∆H = 38.05) and 0.4% NaCl (∆H = 12.21) compared to changes in erythrocyte hemolysis in 0.9% NaCl (Figure S1b).

Rhesus-negative factor erythrocytes showed stronger changes in the release of hemoglobin from cells in media containing 0.85%, 0.45%, 0.4% and 0.3% NaCl compared to those in Rhesus-positive erythrocytes. A two-fold increase in hemolysis was observed from erythrocytes with a Rh-negative factor in 0.3% NaCl in contrast to that in erythrocytes with an Rh-positive factor.

Hematin (80 µM) induced changes in hemoglobin release from Rh-negative and Rh-positive cells in 0.3% NaCl, with the increase in hemolysis being more pronounced in Rh-negative cells.

Pooled IgG (10 mg/mL) caused strong changes in hemoglobin release from Rh-positive erythrocytes in 0.3% NaCl, and changes in Hb release from Rh-negative cells were recorded in Rh-negative erythrocytes at 0.3% and 0.4% NaCl.

Treatment with pooled IgG and hematin did not lead to changes in the hemolysis of erythrocytes with a Rhesus-positive factor in the studied concentrations of NaCl, in contrast to their effect on erythrocytes with a Rhesus-negative factor in 0.3% and 0.4% NaCl, where strong changes in the Hb release were observed.

Preincubation of pooled IgG with hematin did not cause a change in the hemolysis of Rh-positive erythrocytes suspended in all NaCl concentrations tested, in contrast to the observed increase in Hb release from Rh-negative erythrocytes placed in 0.3% NaCl. Therefore, the preincubation of pooled IgG and hematin alters the reduction in the Hb release from erythrocytes with the Rh-negative factor and does not play a role in the action of this combination of biomacromolecules on erythrocytes with an Rh-positive factor (Figure S1).

DMSO caused strong changes in the hemolysis of Rh-positive erythrocytes in 0.3% NaCl and to a greater extent in cells suspended in 0.4% NaCl and 0.6% NaCl in contrast to the increase in hemoglobin release from Rh-negative erythrocytes at 0.3% and 0.4% NaCl.

Melittin at concentrations of 0.5 µg/mL, 2 µg/mL and 5 µg/mL in the presence of pooled IgG did not cause changes in the release of hemoglobin from erythrocytes with the Rh-positive factor at all tested concentrations of NaCl, except for the concentration of “IgG + 0.2 µg/mL melittin”, where an increase in hemolysis was observed in 0.3% NaCl and to a lesser extent in 0.4% NaCl.

### 3.2. Acid—Hemolytic Stability of Erythrocyte Membranes

The extensive research of Gitelzon, Terskov, Leonova and Gomyazkova clearly shows that the resistance of erythrocyte membranes is a function of their age and their physiological state. In the normal state of the individual, this distribution is a function of their age [22,36]. An attempt was made to apply the method of erythrograms to characterize the dynamics of the erythron in the presence of biomacromolecules, using the results of the method of dispersion analysis [37]. In the studies of Terskov and Gitelzon, it is stated that the erythrogram reflects the age composition of the erythrocytes. Then, based on the forms of the erythrogram in the norm, the speed with which the erythrocyte moves from one state to another can be determined [21]. The main points that characterize changes in erythrocytes in terms of resistance are the following: (a) the enhancement of the right shoulder of the erythrogram from the normal position corresponds to the increased percentage of young cells in the blood and speaks of a regenerative process; (b) the stretching of the right shoulder of the erythrogram more than the norm indicates the release of abnormally high-resistance erythrocytes into the blood; (c) the erythrogram possessing two maxima corresponds to the presence of two groups of erythrocytes with very different properties and appears after deep disturbances of the equilibrium state of the blood system; (d) the increase in the left shoulder of the erythrograms corresponds to the relative increase of old erythrocytes. These studies can be used to characterize the state of the erythron in pathology, which is important for observations in the clinics and in physiological experiments. The erythron is a concept related to a change in the dynamic equilibrium of the blood system along with the change in cells in diameter, their physicochemical properties and biochemical composition [37].

#### 3.2.1. Acid Resistance of Erythrocytes with Rhesus-Positive Factor

The acid resistance of the control sample was characterized by a sharp peak at 210 s (value 55.91), and the process of increasing acid resistance started at 150 s (value 1.65) and ended after the peak at 270 s (value 3.95). There was a lag phase of 0–150 s of changes in partial hemolysis in the range of 0–5.27 and 1.65, which was associated with the acid resistance of erythrocytes. Young erythrocytes are represented in the erythrogram in the time region from 210 s to 300 s (value 0) (Figure S2A).

When erythrocytes were treated with 10 mg/mL pooled IgG, a slight increase in acid resistance (value 5.93) was observed at 330 s, indicating that the administered pooled IgG decreased acid resistance by about 11 times compared to the control level.

Hematin (80 µM) affected both old and young erythrocytes, peaking at 180 s (value 21.23), which spanned the range of 90 s (16.09) and 150 s (value 18.11). After 210 s, hematin caused a steep and gradual decline until 270 s (value 0.67), assuming that they are represented by young erythrocytes.

Dimethyl sulfoxide (80 µM), in which hematin was dissolved, shifted the acid erythrogram to the right shoulder and peaked at 270 s (value 28.52); then, at 360 s, it represented all the hemolyzed cells. Old erythrocytes were not affected by DMSO in the period 0–180 s (2.80).

With the administration of pooled IgG and hematin, the right part of the erythrogram became more stretched, which is an indicator of the activation of hemolyzed young cells. The acid erythrogram did not change until 210 s (value 1.79) and then began a slow rise until 480 s (value 15.44) and then a slow decline until 600 s (value 3.67).

The preincubated (IgG and hematin)* showed a similar curve, reaching a maximum acid enhancement at 360 s (value 8.10) and up to 450 s (value 8.94) and then gradually decreasing until 600 s (partial hemolysis value 3.67).

From the recorded partial hemolysis of erythrocytes in the presence of pooled IgG and hematin, as well as pre-incubated (IgG and hematin)*, it can be seen that there was no difference in the acid resistance of erythrocytes after treatment with the indicated agents (Figure S2A).

The lower concentrations of melittin were characterized by a slight increase in the partial hemolysis peak at 60 s (value 6.56 when erythrocytes were treated with 0.2 µg/mL melittin in the suspension medium) and the peak at 30 s (value 9.88) in the presence of 0.5 µg/mL Mt. No changes in partial hemolysis were observed from 180 s (value 1.58) when exposed to pooled IgG and 0.2 µg/mL Mt and at 120 s (value 1.28) after treatment of the erythrocyte suspension with pooled IgG with 0.5 µg/mL Mt. This was followed by a slight increase in the partial hemolysis of young erythrocytes in the presence of pooled IgG and 0.2 µg/mL Mt up to 570 s (value 10.50) and a sharp decrease until 600 s (zero value of partial hemolysis). Pooled IgG in the presence of 0.5 µg/mL Mt resulted in a small peak at 510 s (value 10.46) and a slight decline to partial hemolysis at 600 s (value 6.97) (Figure S2B).

Pooled IgG and 2 µg/mL Mt produced a lower acid resistance of old erythrocytes at 90 s (value 10.28), a decrease at 180 s (value 1.58) and a very weak and slow rise until 600 s (value 11.60).

Erythrocytes treated with pooled IgG and 5 µg/mL Mt were characterized by a lower peak at 90 s (value 6.33) concerning their effect on old erythrocytes, a slight decrease at 210 s (value 1.24) and a slight increase until 330 s (value 9.73), which slightly changed until 600 s (value 4.75).

Melittin as a disruptive agent to the erythrocyte membrane in combination with pooled IgG affected old erythrocytes, as well as young ones, differing from the acid resistance of erythrocytes treated with pooled IgG alone (Figure S2B).

#### 3.2.2. Acid Resistance of Erythrocyte Membranes with Rhesus-Negative Factor

A sharp peak was recorded in the control sample at 270 s (value 29.31) after no changes in partial hemolysis from time zero to 180 s (value 1.12), after which a sharp decline was observed until 450 s (value 0.08) (Figure S2C).

The acid resistance of Rh-negative erythrocytes was shifted by 60 s to the longer onset times of partial hemolysis relative to that of Rh-positive erythrocyte controls, with the lag phase being 30 s longer in Rh-negative erythrocytes.

Hematin-treated erythrocytes had a peak at 270 s (value 26.47) after a lag phase of low, insignificant changes in partial hemolysis from 0–180 s, followed by a bell-shaped increase in ∆H% from 240 s (value 25.06) up to 270 s. After reaching the peak, there was a steep decline until 420 s and no change in partial hemolysis until 600 s.

The acid resistance of Rh-negative erythrocytes was shifted 90 s to the right shoulder of the acid erythrogram compared with that of Rh-positive erythrocytes. Hematin did not significantly alter the acid resistance compared to values of control erythrocytes not treated with hematin.

Pooled IgG caused strong changes in the acid resistance of erythrocytes with a Rh-negative factor, which were expressed in a shift of the acid erythrogram to the left, in 180 s (value 25.70), followed by a gradual decrease in partial hemolysis up to 390 s (value 0.15). The observed lag phase showed slight variations from 0–90 s (values of 3.71–1%) (Figure S2C).

The shift in acid resistance in the presence of 10 mg/mL of pooled IgG from Rh-negative erythrocytes to the left side of the acid erythrogram suggested that the pooled IgG affected old erythrocytes from the erythrocyte suspension in contrast to the partial hemolysis of those treated with pooled IgG erythrocyte membranes with a Rhesus-positive factor. There was a difference in acid resistance between Rh-positive and Rh-negative erythrocytes, indicating the involvement of old Rh-negative erythrocytes and a lesser change in the partial hemolysis of Rh-positive erythrocytes.

When Rh-negative erythrocytes were treated with pooled IgG and hematin, a peak was observed at 270 s (value 21.32) after a steep increase in acid resistance at 120 s (value 2.18) and a lag phase of 0 s (value 3.71) to 90 s (value 1.29). After reaching the peak of partial hemolysis, there was a gradual decrease in acid erythrogram values until 390 s (value 0.89) and no change in ΔH% until 600 s of registration (Figure S2C).

Pooled IgG and hematin applied to the treatment of erythrocytes with the Rh-negative factor was shifted to the left of the acid erythrogram, in contrast to the acid resistance of Rh-positive erythrocytes, which was shifted to the right of the acid erythrogram. Therefore, treatment with pooled IgG and hematin affected old Rh-negative erythrocytes, and in Rh-positive erythrocytes, it led to changes in the acid resistance of young erythrocyte membranes.

The pre-incubated “IgG and hematin” preparation resulted in the same changes in the acid enhancement of Rh-negative erythrocytes as observed when the erythrocytes were treated with pooled immunoglobulin G and hematin. The same value of the acid resistance peak and practically the same drop in the acid erythrogram up to 390 s, as well as no changes in the partial hemolysis of erythrocytes with a Rhesus-negative factor up to 600 s of registration, were found.

No changes were observed in the acid resistance of erythrocytes with the Rh-negative factor in the presence of pooled IgG and hematin, as well as after exposure to pre-incubated pooled IgG with hematin.

Changes in the partial hemolysis of Rh-negative erythrocytes were shifted to the left side of the acid erythrogram after treatment with pooled IgG and hematin, as well as pre-incubated pooled IgG with hematin, in contrast to the acid resistance of Rh-positive erythrocytes, for which changes affected the right side of acid erythrogram and young erythrocytes.

A peak was recorded at 270 s (value 24.09) of the acid erythrogram of Rh-negative erythrocytes after treatment with DMSO, which stood below the control and hematin peaks. This peak was reached after a steep rise in the acid resistance of Rh-negative erythrocytes after a longer lag phase of minor changes in partial hemolysis between 0 s (value 1.60) and 150 s (value 0.76). The steep decline in the acid resistance of DMSO-treated Rh-negative erythrocytes was similar to the steep decline in partial hemolysis in the presence of the untreated control or hematin-treated sample, respectively, reaching a value of 0.67 at 420 s, after which changes in the partial hemolysis of erythrocytes with the Rh-negative factor were observed up to 600 s of registration (Figure S2C).

No significant differences were observed in the acid erythrograms of Rh-positive or Rh-negative erythrocytes treated with DMSO, with the addition that the peak of partial hemolysis of Rh-positive erythrocytes was 4 units higher (value 28%) than the peak of erythrocytes with the Rh-negative factor (value 24%).

A broad plateau was observed in the peak acid resistance of Rh-negative erythrocytes in the presence of pooled IgG and 0.2 µg/mL melittin, from partial hemolysis at 240 s (value 14.0) to a value of 13.42 at 300 s, after which a gradual decline in partial hemolysis was recorded in 570 s to (value 0.24). The observed lag-phase changes in partial hemolysis at 30 s (value 1.14) to that at 90 s were without significant changes (Figure S2D).

The acid resistance of Rh-negative erythrocytes after treatment with pooled IgG and 0.2 µg/mL melittin affected old erythrocytes at 210 s and to a lesser extent the partial hemolysis of control erythrocytes not treated with IgG and the corresponding concentration of melittin. The partial hemolysis of Rh-negative erythrocytes differed strongly from the acid enhancement of Rh-positive erythrocytes, where treatment with pooled IgG and 0.2 µg/mL melittin affected young erythrocytes and was observed at late recording times (570–600 s).

The registration of a steep rise in the acid resistance of Rh-negative erythrocytes after treatment of the erythrocyte suspension with pooled IgG and 0.5 µg/mL melittin in 210 s (value 21.12) and a gradual decrease until 570 s (value 0.24) and 600 s (value 0%) was observed. The lag phase was short, and its duration was from 0 to 90 s before the rise in the acidic erythrogram.

Acid resistance of Rh-negative erythrocytes in the presence of pooled IgG and 0.5 µg/mL melittin affected old erythrocytes with the highest changes in partial hemolysis at 210 s. Similar changes in acid resistance were observed in the presence of 0.5 µg/mL melittin at 180–240 s of recording, suggesting a protective effect of pooled IgG in the presence of the upper concentration of melittin as a membrane-disruptive agent.

The acid resistance of Rh-negative erythrocytes in the presence of 0.5 µg/mL melittin differed from the acid resistance of Rh-positive erythrocytes in the time and magnitude of change of partial hemolysis in the entire time interval. The acid resistance of Rh-negative erythrocytes in the presence of pooled IgG and 0.5 µg/mL melittin affected old erythrocytes, and in Rh-positive erythrocytes, it caused weak changes in partial hemolysis at later times of registration of acid resistance (510–600 s) and affected young erythrocytes (Figure S2D).

After the short lag phase with weak changes in the acid resistance of erythrocytes with the Rh-negative factor after treatment with pooled IgG and 2 µg/mL melittin of 3–1% in the time interval 0–120 s, a steep rise in partial hemolysis was observed at 420–600 s from 1.0% to 0.04%, respectively.

The acid resistance of Rh-negative erythrocytes in the presence of pooled IgG and 2 µg/mL melittin approached the acid resistance of untreated control erythrocyte membranes, affecting old erythrocytes, in contrast to the observed slight changes in the partial hemolysis of erythrocytes with the Rh-positive factor after treatment with pooled IgG and 2 µg/mL melittin, affecting young erythrocytes at late registration times (toward 600 s) (Figure S2D).

A peak was recorded in the acid resistance of Rh-negative erythrocytes after treatment with pooled IgG and 5 µg/mL melittin at 210 s (value 19.59) and 240 s (value 19.43), followed by an exponential decline until 390 s (value 1.47) and a lack of changes in partial hemolysis in the time interval of registration from 420–600 s.

The acid resistance of Rh-negative erythrocytes in the presence of pooled IgG and 5 µg/mL melittin affected old erythrocytes at shorter recording times, in contrast to the partial hemolysis of Rh-positive erythrocytes, where weak changes in acid-hemolytic stability were observed in young erythrocytes at the later registration times (from 330 s to 600 s) (Figure S2D).

### 3.3. Measurements of Proton Transport

#### 3.3.1. Membrane Transport in Erythrocytes (Rhesus-Positive Factor)

The investigated biomacromolecules suppressed the release of protons in the extracellular environment of erythrocytes with a Rhesus-positive factor compared to that in untreated erythrocyte membranes as follows: pooled IgG > hematin ≈ DMSO (Figure S3a).

Pooled IgG and hematin led to the rapid release of protons into the extracellular environment of erythrocytes with the Rh-positive factor for up to 20 s, after which there was a rapid decrease in the process of proton release up to 80 s. This was followed by alkalinization of the suspension medium in time after 80 s and reaching a plateau in the release of alkaline ions in 600 s (Figure S3a).

The pre-incubated sample of pooled IgG with hematin caused a lower change in ∆pH% in contrast to that with pooled IgG and hematin, but the process of the reversal of ion fluxes through the membrane after the peak reached at 20 s was recorded until 60 s, after which a process of alkalinization of the environment, similar to that of the action of pooled IgG and hematin, was observed (Figure S3a).

Therefore, a difference was observed in the kinetic curves of Rh-positive erythrocytes treated with pooled IgG and hematin and erythrocytes after exposure to the pre-incubated (pooled IgG and hematin)*. The peak at 20 s in the pre-incubated sample of pooled IgG and hematin was lower, but stopped with practically the same degree of alkalinization of the suspension medium at 600 s.

There was an overlap of the membrane transport curves (of ∆pH changes) from erythrocytes with a Rh-positive factor in the presence of hematin and DMSO. Similar to the observed kinetics was the curve of changes in ∆pH in the presence of pooled IgG and 0.2 µg/mL melittin and IgG and 2 µg/mL melittin (Figure S3a).

Pooled IgG and 0.5 µg/mL melittin had the strongest influence on the alkalinization of the erythrocyte suspension (Rh-positive factor), where the values reached resembled those of the action of pooled IgG and hematin, as well as the pre-incubated sample of pooled IgG with hematin (Figure S3a).

The concentration of 5 µg/mL melittin in the presence of pooled IgG had no effect on membrane transport, where the highest ∆pH value was 1% at 20 s, after which it maintained zero change in ∆pH from 80–600 s, failing to alkalinize the suspension medium (Figure S3a).

#### 3.3.2. Membrane Transport in Erythrocytes (Rhesus-Negative Factor)

The observed changes in the kinetics of Rh-negative erythrocytes were associated with a slight increase in H^+^ proton efflux in the extracellular space in 20 s, after which a slow decline was registered up to 40 s to a zero level of changes in ∆pH and a gradual alkalinization of the suspension medium, similar to an exponentially similar decline reaching maximal changes in membrane transport (Figure S3b).

Hematin had a slight influence on the acidification of the environment and the release of protons in the extracellular environment of erythrocytes with the Rh-negative factor.

Pooled IgG caused an increase in proton efflux (H^+^ uptake) from Rh-negative erythrocytes at 20 s, causing almost the same changes in membrane transport up to 600 s.

The action of pooled IgG and hematin on erythrocytes with a Rhesus-negative factor was characterized by an increase in the release of protons in the extracellular environment of erythrocytes, and its changes between the peak in 20 s and the time period up to 600 s did not differ significantly (Figure S3b).

The behavior of pre-incubated pooled IgG with hematin was radically different, which was characterized by a steep peak at 20 s, after which the course of changes in ∆pH decreased exponentially until 280 s and changed slightly until 600 s (Figure S3b).

Therefore, there was a large difference in the observed peaks of the ∆pH increase in the Rh-negative erythrocyte membrane, with pre-incubated pooled IgG with hematin being significantly higher than the peak after treatment with pooled IgG and hematin at the initial and short times of the kinetics of ∆pH changes. While the changes in ∆pH transport remained at the same level in the case of pooled IgG and hematin treatment, those of the pre-incubated pooled IgG and hematin samples decreased and spanned the middle and late times of the measurement.

Pooled IgG in the presence of 0.2 µg/mL melittin and pooled IgG and 2 µg/mL melittin caused maximal changes in proton release in the suspension medium of Rh-negative erythrocytes, followed by a steep decline and the suppression of hydrogen ion release for up to 200 s, and these changes were preserved for up to 600 s (Figure S3b).

Pooled IgG in the presence of 0.5 µg/mL melittin showed a steep peak at 20 s and then slowly decreased to 140 s. This effect was related to the release of protons in the medium outside the erythrocytes with the Rh-negative factor, and after 20 s, a reduction in the release of protons in the suspension medium was observed until 140 s, and slight alkalinization of the suspension medium was observed until 600 s (Figure S3b).

The effect of pooled IgG and 5 µg/mL melittin was characterized by a peak at 20 s, peaking after exposure to pooled IgG and hematin. The erythrogram had a steep decline until 60 s, starting to alkalinize the erythrocyte suspension (Rhesus-negative factor) until 220 s, after which changes in ∆pH values did not change significantly until 600 s, and a plateau was noted in reaching these changes (Figure S3b).

### 3.4. Electrokinetic Properties of Erythrocytes in the Presence of pooled Immunoglobulin G, Hematin and Melittin

The electrokinetic properties of the erythrocyte membrane provide information about the surface characteristics of the cell, including their electrophoretic mobility, zeta potential and surface electrical charge. The electrokinetic potential is a measure of cell stability in the erythrocyte suspension without and after treatment with biomacromolecules.

**Figure 2:**
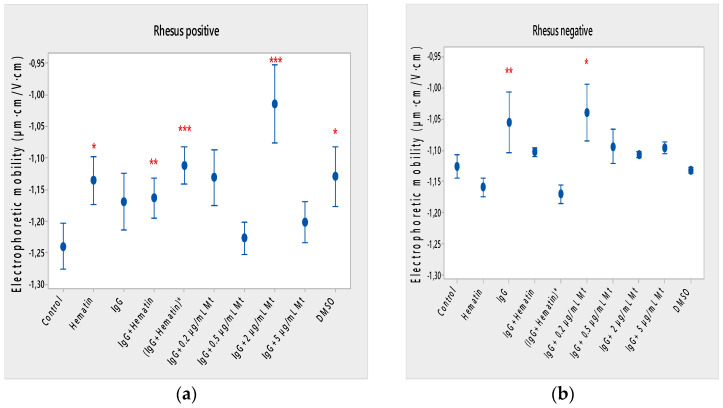
Effect of fixed concentrations of pooled immunoglobulin G (10 mg/mL), hematin (80 µM) and melittin (0.2 µg/mL Mt; 0.5 µg/mL Mt; 2 µg/mL Mt and 5 µg/mL Mt) treatments on electrophoretic mobility (EPM) (average value ± SD) of erythrocytes (**a**) with a Rhesus-positive factor and (**b**) Rhesus-negative factor suspended in PBS, pH 7.4. The pre-incubated sample of pooled immunoglobulin G (10 mg/mL) with hematin (80 µM) is indicated by an asterisk on the abscissa. Erythrocytes were treated with biomacromolecules for 1 h at 37 °C. Electrophoretic mobility was performed at 25 ± 0.1 °C in phosphate buffered saline (PBS), pH 7.4. Each value was the mean ± SD of three independent preparations. *** *p* < 0.001; ** *p* < 0.01; * *p* < 0.05 compared to the untreated control and compared to DMSO (80 µM)-treated control.

**Figure 3:**
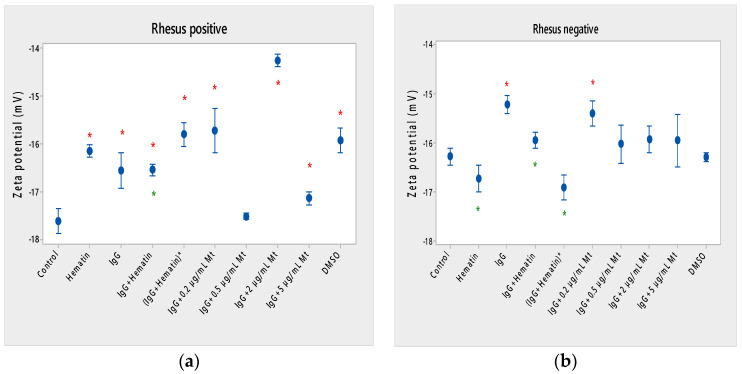
Zeta potential of human erythrocytes (**a**) with a Rhesus-positive factor and (**b**) Rhesus- negative factor upon fixed concentrations of pooled immunoglobulin G (10 mg/mL), hematin (80 µM) and melittin (0.2 µg/mL Mt; 0.5 µg/mL Mt; 2 µg/mL Mt and 5 µg/mL Mt) treatments. The pre-incubated sample of pooled immunoglobulin G (10 mg/mL) with hematin (80 µM) is indicated by an asterisk on the abscissa. The medium contained phosphate buffered saline (PBS), pH 7.4. Erythrocytes were treated with biomacromolecules for 1 h at 37 °C. Electrokinetic measurements were performed at 25 °C in PBS, pH 7.4. Each value is the mean ± SD of three independent preparations. * *p* < 0.05, compared to the untreated control (red asterisks) and compared to DMSO (80 µM)-treated control (green asterisks).

**Figure 4:**
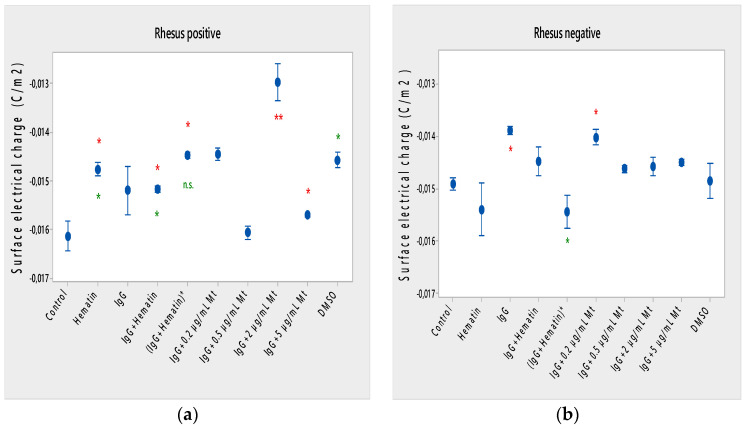
Surface electrical charge of erythrocytes (**a**) with a Rhesus-positive factor and (**b**) Rhesus- negative factor in the presence of pooled immunoglobulin G (10 mg/mL), hematin (80 µM) and melittin (0.2 µg/mL Mt; 0.5 µg/mL Mt; 2 µg/mL Mt and 5 µg/mL Mt) in suspending medium of PBS, pH 7.4. The pre-incubated sample of pooled immunoglobulin G (10 mg/mL) with hematin (80 µM) is indicated by an asterisk on the abscissa. Erythrocytes were treated with biomacromolecules for 1 h at 37 °C. Surface electrical charge measurements were performed at 25 °C in PBS, pH 7.4. Each value is the mean ± SD of three independent preparations. * *p* < 0.05 compared to the untreated control (red asterisks) and compared to DMSO (80 µM)-treated sample (green asterisks).

Hematin decreased the electrophoretic mobility of erythrocytes with a Rhesus-positive factor (** *p* = 0.008) (Figure 2a), decreased the negative values of the zeta potential by 1.5 mV compared to the control (Figure 3a) and reduced the surface electrical charge of the cells by about 6% compared to the charge values of the control erythrocytes (Figure 4a). Hematin did not significantly affect the electrokinetic properties of erythrocytes with a Rhesus-negative factor in comparison to those of the untreated control, but significantly decreased the zeta potential of cells compared to that of the DMSO-treated control (* *p* < 0.05).

Pooled IgG did not significantly affect the electrophoretic mobility, zeta potential and surface electric charge of erythrocytes with the Rh-positive factor. Pooled IgG (10 mg/mL) significantly reduced the electrophoretic mobility of erythrocytes with the Rh-negative factor (Figure 2b). Pooled IgG reduced the zeta potential of the cells with the Rh-positive (Figure 3a) (* *p* < 0.05) and Rh-negative factor by about 1 mV (Figure 3b) (* *p* < 0.05), decreasing the amount of negatively charged groups on the surface of the Rh-negative factor erythrocyte membrane by 10.5% compared to control values (Figure 4b).

The electrophoretic mobility of erythrocytes with a Rhesus-positive factor was decreased in the presence of (IgG+hematin) by 6.5% (* *p* = 0.037) compared to control values of untreated erythrocytes (Figure 2a). The negative electrokinetic (zeta) potential was decreased by 1.1 mV compared to the zeta potential of control erythrocytes without the presence of the above combination of chemical agents compared to control values of untreated erythrocytes, as well as in comparison to the DMSO-treated sample (* *p* < 0.05) in erythrocytes (Rh-negative factor) (Figure 3a,b). The surface electrical charge of erythrocytes with the Rh-positive factor was characterized by a reduction in the amount of negative electric charges exposed on the surface of the cells by 9.5% (Figure 4a).

The pre-incubated (IgG+hematin)* preparation significantly affected the electrokinetic parameters of erythrocytes with a Rhesus-positive factor compared to the untreated control values. A strong decrease in electrophoretic mobility (*** *p* < 0.001) (Figure 2a), a decrease in zeta potential by 1.8 mV (* *p* < 0.05) compared to that in control erythrocytes, was found after treatment with the preincubated pooled IgG with hematin (Figure 3a and Figure 4a). The surface electric charge of Rh-positive erythrocytes also decreased by 12% as a result of the decreased electrokinetic potential and the reduced amount of electric charges on the surface of the erythrocyte membrane (* *p* < 0.05) (Figure 4a). There was an increase in the zeta potential of erythrocytes with an Rh-negative factor upon treatment with preincubated (IgG+Hematin)* compared to that in the DMSO-treated sample (* *p* < 0.05) (Figure 3b).

DMSO decreased the electrophoretic mobility (EPM) of erythrocytes with a Rhesus- positive factor (** *p* < 0.008) by 10% compared to the control values of EPM (Figure 2a). DMSO reduced the negative zeta potential by 1 mV compared to that in the control (Figure 3a) and decreased the negative electrical charges on the surface of erythrocyte membranes by about 11% (Figure 4a). DMSO did not significantly alter the electrophoretic mobility, zeta potential and surface electric charge of Rh-negative erythrocytes compared to those in erythrocytes without DMSO in the suspending medium (Figure 2b, Figure 3b and Figure 4b).

Melittin (0.2 µg/mL) in the presence of 10 mg/mL pooled IgG reduced the electrophoretic mobility of Rh-positive erythrocytes (Figure 2a), an effect that was also observed when exposed to pre-incubated erythrocytes with (IgG+Hematin)* compared to the EPM of control erythrocytes. The electrokinetic potential was strongly reduced by 1.8 mV (*** *p* < 0.001) and the surface charge density was decreased by 12% after treatment with “IgG + 0.2 µg/mL Mt” (Figure 3a and Figure 4a). A significant decrease in the electrophoretic mobility of erythrocytes with a Rhesus-negative factor was found after treatment with “IgG + 0.2 µg/mL melittin” (* *p* < 0.05) compared to the values of control erythrocytes (Figure 2b). The electrokinetic potential of erythrocytes was reduced, and the surface electrical charge was decreased in erythrocytes with a Rhesus-negative factor and after exposure to the above-mentioned concentrations of melittin and IgG (Figure 3b and Figure 4b).

“IgG + 0.5 µg/mL melittin” did not affect the electrophoretic mobility, zeta potential and surface electric charge of erythrocytes with a Rhesus-positive factor (Figure 2b, Figure 3b and Figure 4b).

Significant changes were observed in the electrophoretic mobility of erythrocytes with the Rh-positive factor (*** *p* < 0.001) upon pooled IgG + 2 µg/mL melittin treatment, which was decreased compared to the control EPM (Figure 2a). The zeta potential of Rh-positive erythrocytes treated with “IgG + 2 µg/mL melittin” demonstrated a strong decrease of 3.4 mV compared to that of the control (* *p* < 0.05) (Figure 3b). “IgG + 2 µg/mL melittin” did not cause changes in the electrophoretic mobility, electrokinetic potential and surface electrical charge of Rh-negative erythrocytes compared to those of the control (Figure 2b, Figure 3b and Figure 4b).

An insignificant decrease in the electrophoretic mobility, without changes in the zeta potential and the surface charge density, of erythrocytes with a Rhesus-positive factor was found after exposure to “IgG + 5 µg/mL melittin” (Figure 2b, Figure 3b and Figure 4b). “IgG + 5 µg/mL melittin” did not affect the electrophoretic mobility, zeta potential and surface electric charge of erythrocytes with the Rh-negative factor compared to the control values without these biomacromolecules.

### 3.5. Lipid Peroxidation of Erythrocytes in the Presence of the pooled Immunoglobulin G, Hematin and Melittin

We studied the hydrogen peroxide content at the plasma membrane of erythrocytes with Rhesus-positive and Rhesus-negative factors upon biomacromolecule treatments because the end products of lipid peroxidation through TBARS represent an assay for oxidative damage. Hydrogen peroxide (H_2_O_2_) is produced endogenously in a number of cellular compartments, including at the plasma membrane, where it can play divergent roles as a second messenger or pathological toxic substances [12,27]. Malonaldehyde (MDA) is the most commonly used TBARS corresponding to a marker of oxidative stress.

**Figure 5:**
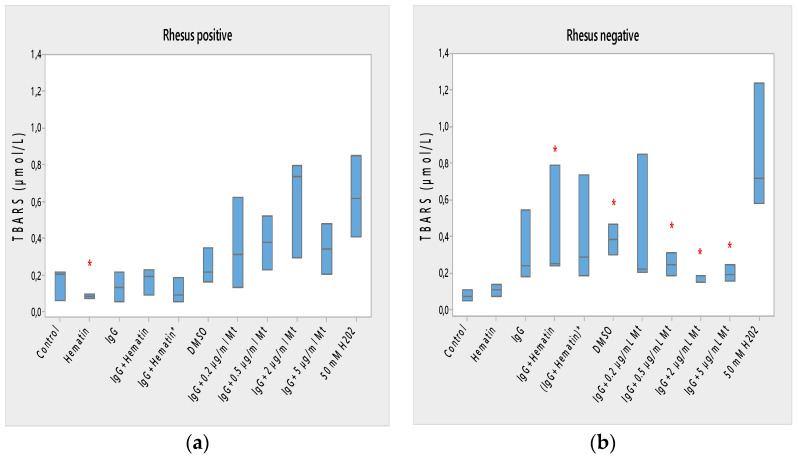
Lipid peroxidation in erythrocytes (**a**) with the Rh-positive and (**b**) Rh-negative factor as a function of fixed concentrations of pooled immunoglobulin G (10 mg/mL), hematin (80 µM) and melittin (0.2 µg/mL Mt; 0.5 µg/mL Mt; 2 µg/mL Mt and 5 µg/mL Mt); erythrocytes were treated with biomacromolecules for 1 h at 37 °C. The pre-incubated sample of pooled immunoglobulin G (10 mg/mL) with hematin (80 µM) is indicated by an asterisk on the abscissa. Lipid peroxidation measurements were performed at 37 °C in phosphate buffered saline (PBS, 2 mM NaN_3_), pH 7.4. Each value is the mean ± SD of three independent preparations. * *p* < 0.05 compared to the untreated control (red asterisks). There were no identified changes in sample values compared to those of the DMSO-treated control. Second control samples contained DMSO (80 µM). The positive control of 50 mM H_2_O_2_ represents the maximal value of TBARS products (incubation of erythrocytes in the presence of 50 mM H_2_O_2_ for 1 h at 37 °C).

Lipid peroxidation of Rh-positive erythrocytes was decreased after exposure to hematin due to the reduced content of TBARS products (* *p* < 0.05) and the decreased oxidation status of Rh-positive factor cells (Figure 5a). Hematin did not significantly affect the formation of lipid peroxidation products in the membrane of erythrocytes with the Rh-negative factor (Figure 5b).

Pooled IgG did not affect the lipid peroxidation of Rh-positive erythrocytes, indicating that secondary lipid peroxidation products are not formed by erythrocytes to a significant extent compared to control ones (Figure 5a). Pooled IgG did not affect the lipid peroxidation of Rh-negative erythrocytes compared with control values (Figure 5b). The combination of (IgG+hematin) did not affect the formation of secondary products of lipid peroxidation in erythrocytes with a Rhesus-positive factor (Figure 5a). An increase in lipid peroxidation in erythrocytes with a Rhesus-negative factor was found as a result of an increase in the formed TBARS products after treatment with pooled IgG and hematin (IgG+hematin) (Figure 5b).

The preincubated (IgG+hematin)* did not change the amount of TBARS products in erythrocyte membranes with a Rhesus-positive factor, as well as in Rh-negative factor cells (Figure 5a,b).

DMSO led to an increase in the lipid peroxidation of erythrocyte membranes with a Rh-negative factor as a result of the increased amount of TBARS products compared to control products (*p* < 0.05) (Figure 5b) without significant changes after treatment in Rh-positive factor erythrocytes.

Melittin (0.2 µg/mL) in the presence of 10 mg/mL pooled IgG did not cause a change in the amount of lipid peroxidation products from Rh-positive erythrocyte membranes (Figure 5a). No changes were found in the amount of secondary products of lipid peroxidation of erythrocytes with a Rh-negative factor after exposure to “IgG + 0.2 µg/mL melittin”, as well as changes in the release of hemoglobin from erythrocytes after exposure to melittin and IgG (Figure 5b).

“IgG + 0.5 µg/mL melittin” increased the products of lipid peroxidation from erythrocyte membranes with a Rhesus-negative factor (* *p* < 0.05) as a result of the formation of an increased concentration of secondary TBARS products from the cells (Figure 5b).

“IgG + 2 µg/mL melittin” increased the lipid peroxidation of erythrocytes with an Rh-negative factor (* *p* < 0.05) (Figure 5b) without alterations to the MDA products in cells with an Rh-positive factor (Figure 5a)

“IgG + 5 µg/mL melittin” did not change the lipid peroxidation of erythrocytes with an Rh-positive factor (Figure 5a) and induced an enhancement of TBARS products in erythrocytes with an Rh-negative factor (* *p* < 0.05, Figure 5b).

### 3.6. Fluorescence Microscopy of Erythrocytes in the Presence of the pooled Immunoglobulin G, Hematin and Melittin

One of the convenient methods for analyzing the morphology of erythrocyte membranes and the change in their shape in the presence of biomacromolecules is the method of fluorescence microscopy [38].

Hematin increased structural and morphological changes on the surface of erythrocytes with a Rhesus-positive factor, where uneven spicules were observed on the outer surface of the membrane, codocytes (target cells), resembling bell-shaped cells, as well as echinocytes. Studies of erythrocyte morphology using the fluorescence microscopy method showed that some of the echinocyte spicules are damaged by hematin (80 µM). “Dull spicules” were noticed, and echinocytes were smaller, with reduced volumes in contrast to codocytes (target cells). A heterogeneous fraction of larger echinocytes and smaller ones was observed, as well as a difference in the shape of erythrocytes with the Rh-negative factor (Figure S4a).

Pooled immunoglobulin G (10 mg/mL) affected the shape of Rh-positive erythrocytes. Echinocyte (crenated) cells with regularly spaced bumps were observed. Different morphologies of erythrocytes with a Rhesus-positive factor were recorded after treatment with pooled IgG and hematin (IgG+hematin): swollen and small echinocytes, codocytes (bell-spaped), stomatocytes, as well as individual types of pyropoikilocyte cells with protrusions in individual poles of the cell.

Different morphologies of erythrocytes with a Rhesus-positive factor were noted after treatment with pooled IgG and hematin (IgG+hematin): swollen and small echinocytes, codocytes (bell-spaped), stomatocytes, as well as individual types of pyropoikilocyte cells with protrusions in individual poles of the cell. A heterogeneous population of erythrocytes with an altered shape was marked. The Rh-negative erythrocyte suspension after treatment with (IgG+hematin) was represented by stomatocytes, codocytes and to lesser extent echinocytes. Individual representatives of pyropoikilocytes were also noticed, which were characterized by extreme variations in size and shape.

Epifluorescence studies showed a heterogeneous population of cells with an altered shape of Rh-positive erythrocytes after treatment with preincubated (IgG+hematin)*. Smaller echinocytes were observed compared to those observed with (IgG+hematin)-treated erythrocyte membranes. Echinocytes in the preincubated sample (IgG+hematin)* had dense luminescent areas on the erythrocyte surface as a result of the interaction of FITC-CoA and the specific areas on the erythrocyte membrane for concanavalin A, which covered a specific area of the cell surface. Thus, a clustering of luminous spicules of echinocytes towards one pole of the cell was marked, affecting a separate area of the membrane surface. Larger codocytes (bell-shaped) and stomatocytes were noted. Fluorescence microscopy studies showed the presence of fewer Rh-negative erythrocytes after treatment with preincubated (IgG+hematin)* and more erythrocyte ghosts surrounded by a luminous halo after the interaction of FITC-CoA with cellular receptors for concanavalin A on the outer surface of the membrane.

Echinocytes, codocytes (bell-shaped), were observed. Separate elliptocytes (pencil-shaped cells) were viewed, characteristic of membrane defects or related to effects of the activity of spectrin and protein 4.1 in cells with a Rhesus-negative factor.

Treatment of Rh-positive erythrocytes with DMSO led to the appearance of burr cells (echinocytes), acantocytes and codocytes with luminous halos as a result of FITC-CoA interactions with receptors on the surfaces of erythrocytes for concanavalin A. When exposed to DMSO, erythrocytes with a Rhesus-negative factor with a different shape were recorded. Echinocytes and codocytes (bell-shaped) were noticed.

Melittin in the presence of 10 mg/mL pooled IgG labeled as “IgG + 0.2 µg/mL Mt” resulted in a heterogeneous population of Rh-positive erythrocytes, with spherocytes observed. Echinocytes and codocytes with unevenly protruding spicules were observed on the outer surface of the cell. Epifluorescence studies showed altered shapes of Rh-negative erythrocytes treated with “IgG + 0.2 µg/mL melittin”. Echinocytes (crenated) with unevenly distributed spicules on the surface of the cell and codocytes were noticed.

Epifluorescence of Rh-positive erythrocytes after exposure to “IgG + 0.5 µg/mL melittin” showed different shapes of erythrocytes, similar to those observed in “IgG + 0.2 µg/mL melittin”-treated cells. Swollen echinocytes with a well-defined luminous halo resulting from the fluorescence of FITC-CoA binding to membrane receptors for concanavalin A were marked, as well as stomatocytes and codocytes. Fluorescence microscopy studies showed different shapes of Rh-negative erythrocytes treated with “IgG + 0.5 µg/mL melittin”. Spherocytes with regularly spaced bumps and erythrocyte ghosts were recorded (Figure S4a).

Fluorescence microscopy of Rh-positive erythrocytes treated with “IgG + 2 µg/mL melittin” showed a different cell shape. Echinocytes and codocytes were observed, which were in lower numbers and with a greater number of ghost cells, which were elongated as elliptocyte (pencil-shaped) membranes with membrane defects, where the spectrin and protein 4.1 could be affected (Figure S4b). Treatment with “IgG + 2 µg/mL melittin” of Rh-negative erythrocytes showed a change in erythrocyte morphology. The oval shape of the erythrocytes prevailed, like an “egg shape”, associated with ovalocytosis and a possible variation in hemoglobin content. The observed changes in oval erythrocytes with a Rhesus-negative factor can provide additional information about the relationship of ovalocytes and elliptocytes with some pathological processes, such as myelodysplastic syndrome, thalassemic syndrome and megaloblastic processes associated with the formation of macrocytes. Ghost cells were noticed, which glowed with thickened luminous streaks at the area of Rh-negative erythrocytes. Ghost cells were rounded and swollen with a highly pronounced luminous halo around the erythrocyte membrane, devoid of hemoglobin and as a result of the binding of FITC-CoA to concanavalin A receptor on “IgG+2 µg/mL melittin”-treated membranes of ghost cells (Figure S4b).

Based on the fluorescence microscopy studies of erythrocytes with a Rhesus-positive factor after treatment with “IgG + 5 µg/mL melittin”, a fraction of codocytes (bell shaped cells) and individual stomatocytes was observed. “IgG + 5 µg/mL melittin” caused the appearance of more ghost cells and fewer erythrocytes in the fluorescence microscopy samples, which were characterized as echinocytes, codocytes (target cells). Ghost cells had a less luminous outline than that of Rh-positive erythrocytes due to the binding of FITC-CoA to membrane receptors for concanavalin A. Elliptocyte-like ghost cells associated with membrane defects on the surface of ghost cells were marked.

### 3.7. Effect of Melittin on the Impedance Characteristics of Model Lipid Membranes

**Figure 6:**
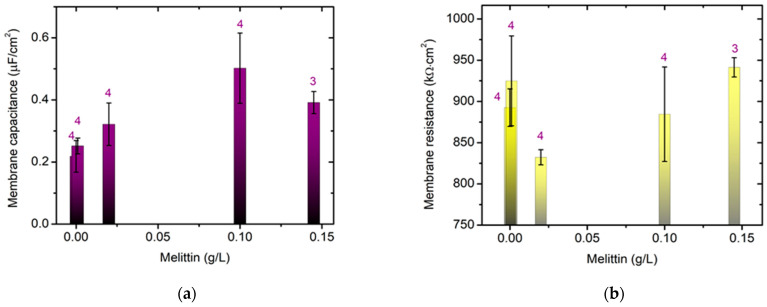
Membrane capacitance (**a**) and membrane resistance (**b**) of egg PC bilayers in 1.25 mM Na_2_HPO_4_/KH_2_PO_4_ buffer (ionic strength of 0.003 mol/L), pH 7.4, in the presence of melittin. The membrane resistance and membrane capacitance are presented as the weighted mean ± SD; the number of independent measurements, with each of them averaged over 10 repetitions, is indicated above the bars.

The electrical capacitance and resistance of suspended egg PC bilayers were investigated via FFT-EIS for non-specific interactions of melittin with the lipid matrix of the membrane. Egg PC bilayer samples formed in 1.25 mM Na_2_HPO_4_/KH_2_PO_4_ buffer were studied as controls. The effect of the lytic peptide was measured in a wide concentration range from 1 µg/mL up to ~0.15 mg/mL, corresponding to ~0.4 µM and ~50 µM, respectively. Melittin is known to be monomeric in low ionic strength buffers to at least a concentration of 0.3 mM [39]. For a given melittin concentration, at least three BLMs were prepared and subjected to impedance measurements, each of them averaged over 10 repetitions. The weighted mean values of the studied electrical parameters of egg PC membranes with the corresponding standard deviations are presented in Figure 6. The fitting of the experimental data with the equivalent model circuit [31] yielded resistance of egg PC bilayers close to the values reported for phosphatidylcholine bilayers, hitherto, $R_{m}$ ~ 10^6^ Ω cm^2^ [13,31,40,41]. The presence of 0.02 g/L melittin in the bulk solution increased the BLM capacitance and more significantly reduced the membrane resistance, as shown in Figure 6. We observed non-monotonic behavior of both parameters at the highest total peptide concentration in the sample, namely 0.145 g/L, corresponding to 50 µM of melittin. This finding suggests the hypothesis of a non-monomeric conformation of the peptide in the bulk phase [39], a hypothesis that needs further exploration.

## 4. Discussion

The open question of how the electrokinetic and other biophysical properties of the membranes behave under pooled IgG, hematin and melittin treatments in terms of dependence on the Rhesus factor of erythrocytes remains unclear, taking into account the effective charges of the biomacromolecules that they exhibit during their interaction with the cell. When the erythrocytes come into contact with the antibodies, they are fixed on the surface of the erythrocyte membranes. In this way, the surfaces of erythrocytes do not carry their own charge, but the charge of globulin molecules, which is much smaller. Therefore, the erythrocyte suspension when treated with pooled IgG is expected to be much more unstable, and aggregation occurs more easily. The results reported here indicate that pooled IgG causes a decrease in the surface electrical charge of erythrocytes with a Rhesus-negative factor, but does not affect erythrocytes with a Rhesus-positive factor. Pooled IgG can be considered a bipolar ion with charge homeostasis in physiological media (PBS, pH 7.4) with an effective Debye–Hückel–Henry charge between −3 and −9 [42], the effective charge being calculated as Z_DHH_ = 7.7 ± 0.2 [43], with the indication that salt and temperature dependence can be included in the calculation of the effective charge of pooled immunoglobulins. Changes in cell morphology in Rh-positive cells include the appearance of echinocytes, codocytes, stomatocytes and “ghost” cells. Pooled IgG (10 mg/mL) leads to a decrease in hematocrit and acid resistance of erythrocytes with a Rhesus-positive factor, but causes an increase in membrane transport, as well as the process of a strong release of hemoglobin in a medium of 0.3% NaCl. The action of pooled IgG on erythrocytes with a Rhesus-negative factor differs from that on erythrocytes with a Rhesus-positive factor, except in the case of lipid peroxidation of cells, where no change in secondary products of lipid peroxidation is registered. Pooled IgG causes the appearance mainly of codocytes, as well as of echinocytes, when examining the morphology of erythrocytes. In erythrocytes with a Rhesus-negative factor, pooled IgG causes a decrease in electrophoretic mobility, electrokinetic potential and surface electrical charge, hematocrit and acid-hemolytic stability of cells. Pooled IgG leads to an increase in membrane transport across erythrocyte membranes with a Rhesus-negative factor, as well as in the release of hemoglobin in a medium of 0.3% NaCl.

Heme acts as a signaling molecule, which suggests the possibility of dynamic and rapid mobilization from different carrier and scavenger proteins [44,45]. The heme-induced activation of the blood coagulation system is suggested as a mechanism for the initiation of thrombotic events under hemolytic conditions [46]. Several reports describe the determination of heme concentrations in different conditions, which range from ~20 to >350 µM as occurring, e.g., in heme-driven pathologies [47]. In our studies, the concentration of 80 µM hematin was used, at which there is weak release of hemoglobin from the cells, but the acid-hemolytic stability is significantly reduced. We believe that the presence of hematin (80 µM) and pooled IgG (10 mg/mL) in the erythrocyte suspension creates a situation where the antibody should appear as a cation, because of the acidification of the environment due to the hematin pKa (strongest acidic), which is 3.68, and pKa (strongest basic), which is 4.96. The physiological charge of hematin is (−2) [48]. Therefore, the action of the preincubated sample of pooled IgG with hematin and of the pooled IgG in the presence of hematin will have a strong effect in decreasing the surface charge of Rh-positive erythrocytes, but will not affect the surface electrical charge of erythrocytes with the Rh-negative factor. Obviously, the preincubation of pooled IgG with hematin at 37 °C, as well as the action of pooled IgG and hematin on erythrocytes with a Rhesus-negative factor, could eliminate changes in the surface electrical charge of the cell in our experimental setup. That is the reason the changes caused by hematin need to be investigated.

In the present study, using erythrocytes with Rhesus-positive and Rhesus-negative factors, the action of pooled IgG (10 mg/mL), hematin (80 µM) and melittin (0.2 and 0.5 µg/mL; 2 and 5 µg/mL) on erythrocyte membranes was studied in detail.

Hematin (80 µM) decreases the lipid peroxidation in the erythrocyte membrane, the electrokinetic properties of erythrocytes with a Rh-positive factor and the acid-hemolytic stability of the cell. Therefore, hematin eliminates the increased amount of radical forms from erythrocytes after treatment with DMSO, in which hematin is dissolved in our studies. Hematin preserves the tendency to decrease the electrophoretic mobility, zeta potential and surface electrical charge of Rh-positive erythrocytes after treatment with DMSO. The direction of action of hematin or DMSO in the treatment of erythrocyte membranes with a Rhesus-positive factor is also similar. In this case, the hematocrit does not change, the release of hemoglobin from erythrocytes is slightly increased, the membrane transport through the membrane is strongly increased and the acid-hemolytic stability of the cells is significantly reduced. Erythrocytes with a Rhesus-positive factor treated with hematin are characterized by the appearance of “ghost” cells, echinocytes and codocytes. In the case of erythrocytes (Rhesus-negative factor), codocytes and spherocytes are observed after treatment with 80 μM hematin. This is dominated by “ghost” cells with a loss of hemoglobin. The ghost cells indicate coagulative necrosis where there is cell death but retainment of cellular architecture. Spherocytes are characteristic of hemolytic anemia. A reduction in free radical forms (TBARS) of erythrocytes with a Rhesus-positive factor after exposure to hematin is observed due to a decreased concentration of malondialdehyde in cells.

The combined effect of pooled IgG and hematin, as well as the preincubation of the individual components, shows its different effect on cell morphology and biophysical parameters of erythrocytes with Rhesus-positive and those with Rhesus-negative factors. More codocytes, echinocytes and pyropoikilocytes are observed in the erythrocyte sample with a Rhesus-positive factor. Most of the codocytes are resistant to osmotic lysis compared to those not treated with pooled IgG and hematin control cells. Confirmation for this is obtained from the results of the release of hemoglobin on erythrocytes with a Rhesus-positive factor, as Hb-release is strongly reduced compared to control values, untreated cells. This effect is related to the increase in the ratio between the surface area and the volume of the cells and the decreased osmotic resistance of the erythrocytes with a Rhesus-positive factor. Treatment with pooled IgG and hematin causes the appearance of stomatocytes, except for codocytes in erythrocytes with the Rh-negative factor. Stomatocytosis are due to alterations in permeability, leading to an increased volume. “IgG+hematin” and preincubated (IgG+Hematin)* results in changes in lipid peroxidation, hematocrit and hemoglobin release from erythrocytes with a Rhesus-negative factor. The sample containing “IgG+hematin” significantly increased TBARS products from lipid peroxidation, in contrast to the absence of such in the pre-incubated form (IgG+Hematin)* on erythrocytes with a Rhesus-negative factor. “IgG and hematin” preparations lead to a strong decrease in the hematocrit of erythrocytes with a Rhesus-negative factor, in contrast to the effect of preincubated (IgG+Hematin)*, where no changes in the volume percentage of the red blood cells in the blood with a Rhesus-negative factor are observed. Rhesus-negative factor erythrocytes treated with “IgG+hematin” cause an increase in Hb release from cells, and those treated with pre-incubated (IgG+hematin)* are not characterized by changes in hemoglobin release from Rhesus-negative factor erythrocytes. It is important to note that erythrocytes with a Rhesus-positive factor are characterized by a lack of changes in lipid peroxidation, hematocrit and the release of hemoglobin from the cell, as well as an increase in membrane transport after incubation with “IgG+hematin” and pre-incubated (IgG+hematin)*. A significant decrease in the electrokinetic parameters of the erythrocytes with a Rhesus-positive factor and of the acid resistance is observed after the treatment of cells with “IgG+hematin” and of the pre-incubated sample of (IgG+hematin)*. The results show that compared to that in the control containing DMSO in the erythrocyte suspension, there is an increase in the zeta potential of Rh-negative erythrocytes in the presence of hematin and no statistically significant changes in the electrokinetic potential of Rh-positive erythrocytes. Treatment with preincubated (IgG and hematin)* in erythrocytes with Rh-positive factor causes the appearance of codocytes, stomatocytes and echinocytes, unlike the presence of many “ghost” cells, codocytes and spherocytes in cells with a Rhesus-negative factor.

Hematin and pooled IgG, as well as the pre-incubated preparation of (IgG+hematin)* mark a slight increase in the negative zeta potential values (* *p* < 0.05) of Rh-negative erythrocytes, which we believe is due to the ability of hematin to influence the conformational changes of the molecule of the pooled IgG and convert it into an IgG/hematin complex capable of causing the exposure of additional negatively charged groups on the surface of Rh-negative erythrocytes. The zeta potential changes are significant, and hematin causes an increase in the negative electrokinetic potential of the membrane when only the hematin molecule is applied in comparison to that of the DMSO-control. It can be seen that the zeta potential of Rh-negative erythrocytes treated with pooled IgG and hematin is slightly decreased (* *p* < 0.05) compared to that of the DMSO-treated control, but the pre-incubated sample of both components show an increase in electrokinetic potential of the erythrocyte membrane with a Rhesus-negative factor.

The kinetics of membrane transport of erythrocytes with the Rh-positive or Rh-negative factor are characterized by an increase in ΔpH changes up to 20 s and the subsequent influx of protons into the interior of erythrocytes from 20–600 s of measurement.

The main action of melittin on erythrocyte membranes is associated with its hemolytic activity [49,50,51,52]. In the present work, the action of melittin on human erythrocytes as a melittin-induced membrane permeability agent with a monomer species [53] is investigated, where the hemolytic action of low concentrations of melittin on cells (Rhesus-positive factor, previous investigations) is established. The emphasis in the present research involving disruptive monomeric melittin-membrane properties has been shifted to the influence of pooled IgG in the presence of fixed concentrations of melittin, where a decrease in the zeta potential of cells under the action of different concentrations of melittin is found.

The effective charge of melittin in isotonic PBS medium, pH 7.4, is calculated as 4.24 [54]. The combined action of melittin and pooled IgG makes the electrokinetic behavior of erythrocytes different in the presence of fixed concentrations of melittin, which is known to increase the electrokinetic potential of human erythrocytes, without the presence of pooled IgG in the erythrocyte suspension [55]. Pooled IgG (10 mg/mL) and melittin caused an increase in lipid peroxidation of Rh-negative erythrocytes except for the sample containing (IgG + 0.2 µg/mL) melittin. The action of pooled IgG and fixed concentrations of melittin does not affect MDA-products in Rh-positive erythrocytes. The fixed concentration of 0.2 µg/mL melittin in the presence of 10 mg/mL pooled IgG leads to a decrease in electrophoretic mobility and a decrease in surface electrical charge due to a decrease in the zeta potential of erythrocytes with a Rhesus-positive factor, via a decrease in the acid-hemolytic stability of cells, an increase in membrane transport of the erythrocyte suspension. Data from fluorescent microscopy confirm the presence of echinocytes, spherocytes and ghost cells in erythrocytes with Rhesus-positive factor, as well as echinocytes in the effect on erythrocytes with a Rhesus-negative factor in the presence of pooled IgG and 0.2 μg/mL melittin. These forms of erythrocytes are characteristic of other concentrations of the effect of pooled IgG and melittin on erythrocytes with the Rh-positive factor. Pooled IgG in the presence of 2 µg/mL melittin induces an increased amount of radical forms of Rh-negative erythrocytes and leads to a decrease in hematocrit and acid resistance, but is associated with an increase in membrane transport across the membrane. However, with the doses of pooled IgG and 2 μg/mL melittin, ovalocytes are observed in cases of exposure to erythrocytes with the Rh-negative factor. The effects of “IgG + 2 µg/mL melittin” or “IgG + 5 µg/mL melittin” preparations on the biophysical parameters of the membrane decrease the surface charge of erythrocytes with a Rhesus-positive factor, but significantly reduce the hematocrit, acid-hemolytic stability of erythrocytes with both Rh factors. Rhesus-positive and Rhesus-negative erythrocytes, after treatment with “IgG + 2 µg/mL melittin” and “IgG + 5 µg/mL melittin”, are characterized by a strong increase in the membrane transport of protons, chloride and hydroxyl ions, which means that pooled IgG in the presence of 2 µg/mL melittin and 5 µg/mL melittin, respectively, increases H^+^/Cl^−^ coexchange and H^+^/OH^−^ transport, reducing the acid resistance of erythrocyte membranes and hematocrit. The additional TBARS products are not detected as a result of incubation with Rh-positive erythrocytes, but lipid peroxidation increases significantly in Rh-negative erythrocytes. When pooled IgG and 0.5 μg/mL melittin act on erythrocytes with a Rhesus-positive factor, stomatocytes are observed, as well as with a dose of impact of pooled IgG and 5 μg/mL melittin on erythrocytes with a Rhesus-negative factor. The different forms of erythrocytes after their impact with biomacromolecules will also lead to different biophysical actions on the membranes of cells with the Rh-positive or Rh-negative factor.

Based on our previous studies on the effect of melittin on the electrokinetic properties of biological membranes, we consider it particularly important to monitor the membrane capacitance of model lipid bilayer membranes in the presence of different concentrations of melittin. The electrochemical impedance study of model lipid bilayers in the presence of melittin yields the membrane electrical capacitance and resistance in low ionic strength media (I = 0.003 mol/L). As expected, at low concentrations, ~0.02 g/L monomeric melittin triggers pore formation [56] resulting in lower specific resistance of the bilayers, as shown in Figure 6b. Benachir and Lafleur [57] investigated the mechanism of interactions of melittin with lipid vesicles and found that melittin leads to a complete and abrupt emptying of the contents of the vesicles. This mechanism obeys the “all or nothing” principle, where melittin acts rapidly within seconds to minutes [57].

In the presence of higher peptide concentrations (~0.15 g/L) in the buffer medium [4,5,58], melittin tetramers form, by means of hydrophobic bonds between the monomers [4,5]. The aggregation of melittin is expected at the higher melittin concentrations, ~0.15 g/L, in the bulk phase [59] at which increased resistance is measured compared to that in untreated lipid bilayers (Figure 6b). Lipid bilayer capacitance is higher in the presence of 0.10 g/L melittin compared to that in the control, which is likely related to bilayer thinning [60] and alterations to the dielectric permittivity of melittin-treated membranes. Thus, the stabilization of the lipid phosphatidylcholine bilayer is characterized also by higher specific capacitance and resistance upon treatment with tetrameric melittin in the presence of 0.15 g/L melittin in the buffer medium and a higher membrane capacity compared to that of the control. The positively charged peptide interacts electrostatically with the membrane. An increase in the surface density of the negative charge of the membrane leads to a decrease in the lytic power of the membrane. The effective charge of melittin is about 2 [61] and is significantly lower than its net electrical charge of 5–6 expected at neutral pH.

The interaction of melittin with the membrane is sensitive to the lipid composition of the bilayer. Lipids with negatively charged heads and cholesterol inhibit the lytic activity of melittin [62,63]. It has been suggested that the primary and secondary structure of a peptide determines its interaction with specific lipid moieties. Even when there is no lytic activity, melittin binds to the membrane. Therefore, the reduced activity of the peptide is not caused by reduced binding to the lipid bilayer.

As the concentration of melittin increases, the membrane capacitance increases, and at 0.15 g/L melittin in the egg–yolk lecithin system, a decrease in the electrical capacitance value is observed compared to that with 0.10 g/L melittin, which could be explained by the thinning of the lipid bilayer thickness observed with the higher concentrations of the cationic peptide [60] and a subsequent decrease in the effect of melittin on membrane capacitance.

The obtained results might be useful in understanding the functional significance of therapeutic antibodies exposed to pro-oxidants, using the biophysical explanation of their action on human erythrocytes [64]. The acquired knowledge can also be used as additional information at the membrane level in unraveling the role of therapeutic immunoglobulin G in its application in inflammatory diseases [65].

## 5. Conclusions

The pooled IgG, hematin, melittin and combinations of these chemical agents change the morphology of erythrocytes from both the Rhesus-positive or Rhesus-negative factors. This is due to effects on the activity of the Band 3 protein, which carries out proton and anion transport through the erythrocyte membrane depending on the Rhesus factor of the erythrocytes. It is necessary to note the important role of the spectrin network in the shear elasticity of the red blood cells, which also exhibits a change in the bending rigidity [65].

The electrokinetic properties of erythrocytes are affected, which are characterized by a reduction in negatively charged surface-exposed groups in the presence of pooled IgG and hematin, as well as its preincubated form, after exposure to erythrocytes with a Rhesus-positive factor. The oxidative status of erythrocytes with a Rhesus-negative factor is also affected, which demonstrates an increase in lipid peroxidation under the influence of pooled IgG and hematin, as well as under the influence of pooled IgG with fixed concentrations of melittin. It is noted that MDA products are affected only under the influence of 80 µM hematin after exposure to erythrocytes with the Rh-positive factor. It is necessary to note the influence of the studied biomacromolecules on the hematocrit of erythrocytes. The hematocrit of erythrocytes with a Rhesus-positive factor is affected only by pooled IgG and its combination with melittin, in contrast to the hematocrit of erythrocytes with a Rhesus-negative factor, where a reduction in the parameter is also observed with hematin, pooled IgG and hematin and pooled IgG and melittin. Thus, we prove that the noticed biomacromolecules in the applied concentrations of impact on the erythrocyte membrane lead to a change in the biophysical parameters of the erythrocytes depending on their Rhesus factor (Rhesus-positive or Rhesus-negative).

Pooled IgG and hematin, as well as the preincubated sample, strongly suppress the acid resistance of young erythrocytes with the Rh-positive factor, but reduce it to a lesser extent in old erythrocytes with an Rh-negative factor. When increasing melittin concentrations to 0.15 g/L, an increase in membrane capacitance of egg yolk PC lipid bilayer is observed, but with a decrease in the membrane resistance, which is associated with the tetrameric form of the peptide affecting the impedance of the model system.

The present studies add to our fundamental knowledge of the action of pooled immunoglobulin G and hematin, as well as pooled immunoglobulin G and melittin, on the human erythrocyte membrane.

## Data Availability

Not applicable.

## Supplementary Materials

The following supporting information can be downloaded at: https://www.mdpi.com/article/10.3390/membranes13030281/s1, Figure S1: Hemolysis changes in erythrocytes (a) with a Rhesus-positive factor (Variant 1) or (b) with Rhesus-negative factor (Variant 1) in the presence of fixed concentrations of Hematin (80 µM) (Variant 2), pooled Immunoglobulin G (10 mg/mL) (Variant 3), IgG+Hematin (Variant 4), preincubated (IgG+Hematin)* (Variant 5), and Melittin (0.2 µg/mL Mt (Variant 6); 0.5 µg/mL Mt (Variant 7); 2 µg/mL Mt (Variant 8) and 5 µg/mL Mt)(Variant 9), after incubation in vitro in different concentrations of NaCl. Figure S2: HCl—Hemolysis changes in erythrocytes with Rhesus-positive factor (A, B) or Rhesus-negative factor (C, D) in the presence of fixed concentrations of pooled Immunoglobulin G (10 mg/mL), Hematin (80 µM) and DMSO (80 µM) and pooled Immunoglobulin G in the presence of Melittin (0.2 µg/mL Mt; 0.5 µg/mL Mt; 2 µg/mL Mt and 5 µg/mL Mt) after incubation in vitro for 1 hour at 37 °C. Figure S3. Extracellular proton concentration (H^+^_ext_) as a function of time (s), calculated from the recorded ∆pH changes induced by sudden jumps of the extracellular proton concentration of erythrocytes (a) (Rh-positive) or (b) (Rh-negative) in Sucrose, salt-free medium (NaOH) with pH 7.4, upon the treatment of fixed concentrations of pooled Immunoglobulin G (10 mg/mL), Hematin (80 µM) and Melittin (0.2 µg/mL Mt; 0.5 µg/mL Mt; 2 µg/mL Mt and 5 µg/mL Mt), respectively. Figure S4. Fluorescence images of human erythrocytes with a Rhesus-positive factor (a) and Rhesus-negative factor (b) without biomacromolecules (control, A) and treated by DMSO (80 µM), (B), by hematin (80 µM, C), by pooled Immunoglobulin G (10 mg/mL, D), by pooled Immunoglobulin G (10 mg/mL) and hematin (80 µM) as (E) or by preincubated sample of pooled Immunoglobulin G (10 mg/mL) and hematin (80 µM) as (F). Fluorescent images for erythrocytes with Rhesus-positive factor or Rhesus-negative factor incubated with pooled Immunoglobulin G (10 mg/mL) and with melittin at 0.2 µg/mL (G); 0.5 µg/mL (H); 2 µg/mL (I) and 5 µg/mL (J) for 60 min.

## Abbreviations

PBS	phosphate buffered saline
FITC	fluorescein isothiocyanate
CoA	concanavalin A
PI	propidium iodide
DMSO	dimethyl sulfoxide
Hct	hematocrit
Hb	hemoglobin
Mt	melittin
IgG	immunoglobulin G
MDA	malondialdehyde
Rh	Rhesus factor
BLM	bilayer lipid membrane
PC	L-α-phosphatidylcholine
FFT-EIS	fast Fourier transform electrochemical impedance spectroscopy
TCA	trichloroacetic acid
TBARS	thiobarbituric acid reactive substances
SD	standard deviation
σ	surface electrical charge (surface charge density)
ζ	zeta (electrokinetic) potential
