# Supplementary material for: Erythrocyte Membrane Biophysical Changes Mediated by Pooled Immunoglobulin G and Hematin: Electrokinetic and Lipid Peroxidation Studies
Source: Membranes (Basel). 2023 Feb 27;13(3):281. doi: 10.3390/membranes13030281 (PMC10056742; doi:10.3390/membranes13030281)
Supplement: Supplementary file 1 [file membranes-13-00281-s001.zip › Supplementary Material_Graphs_membranes-2161721.pptx]

## Slide 1
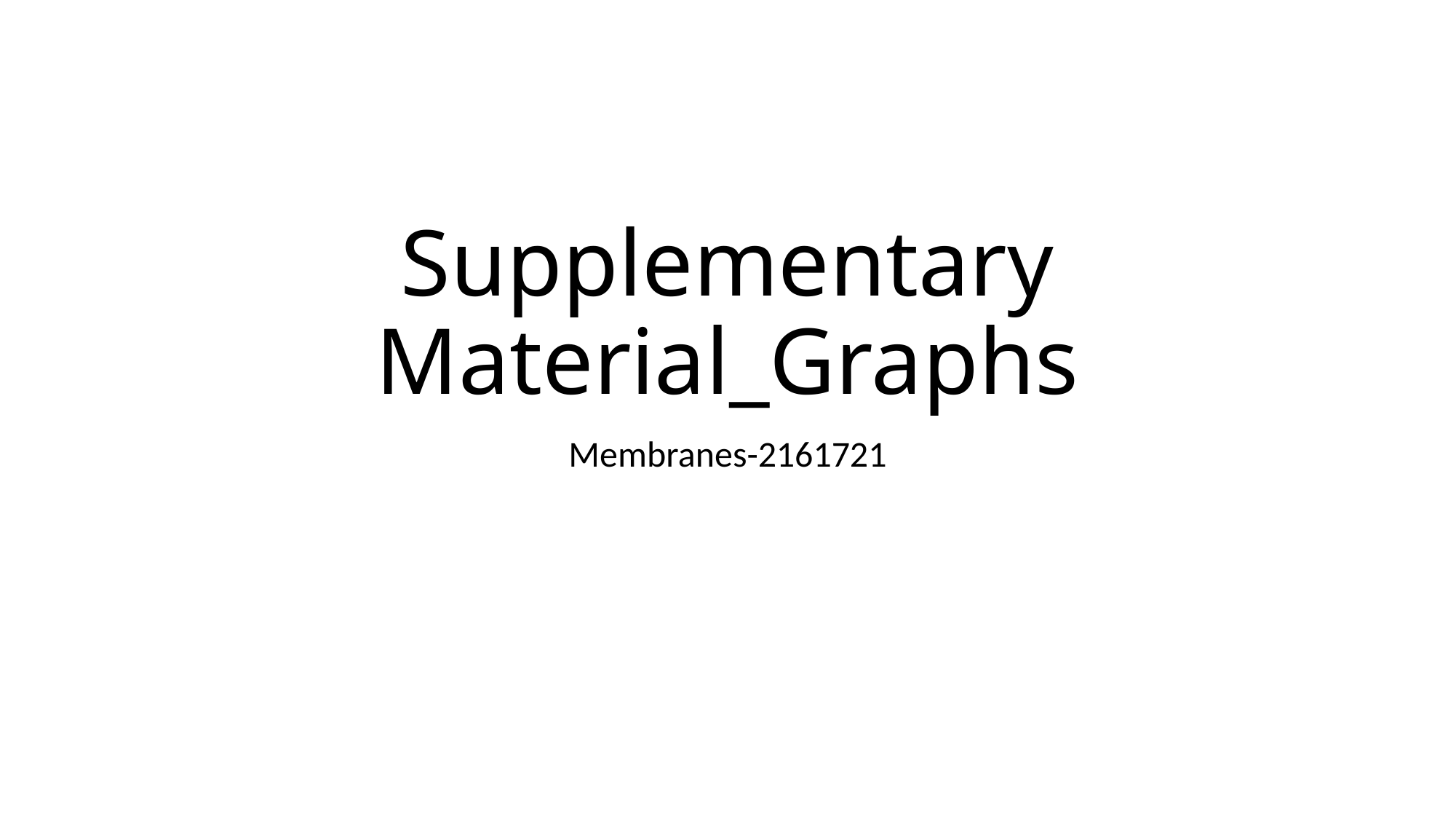

# Supplementary Material_Graphs
Membranes-2161721

## Slide 2
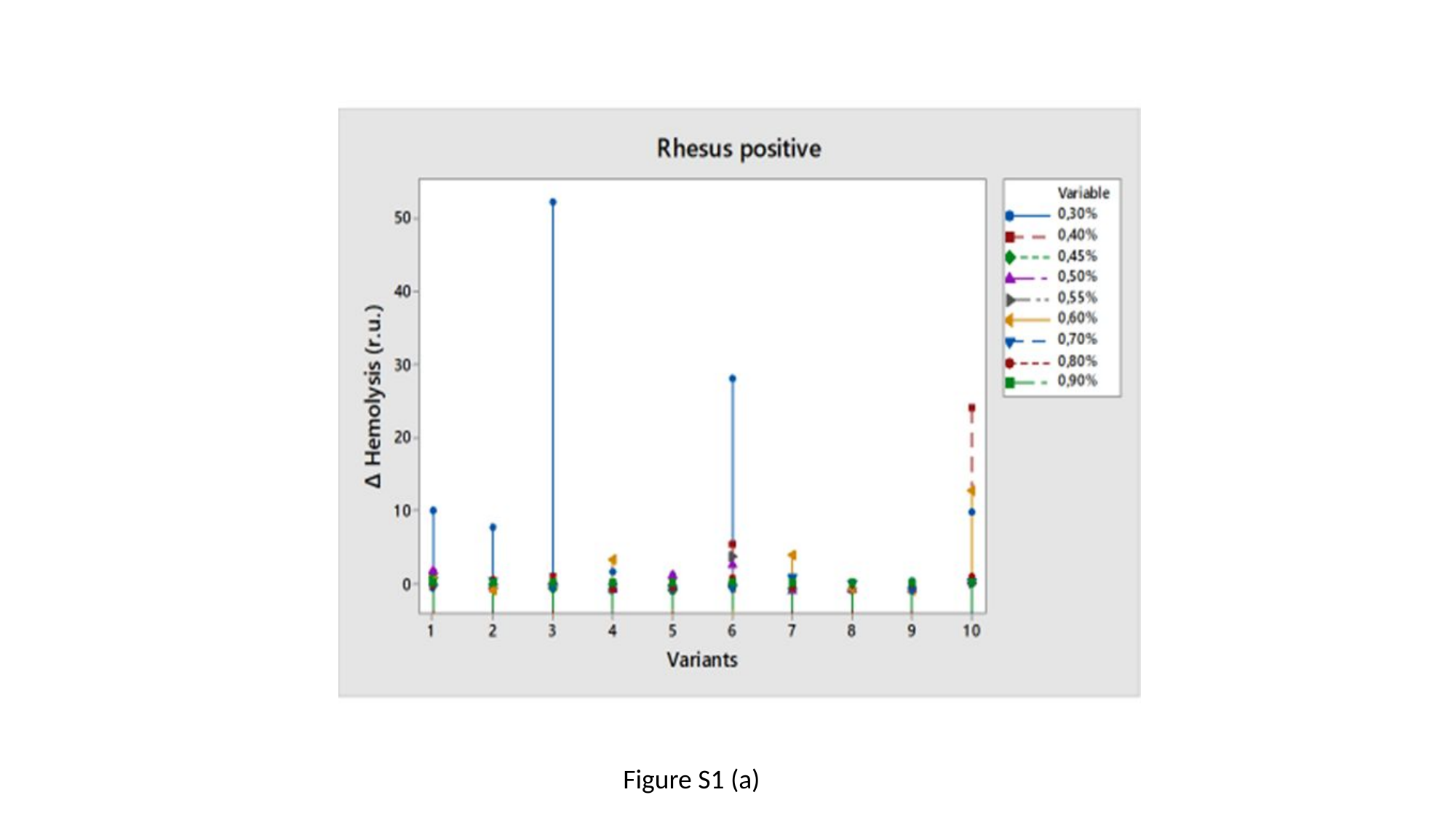

Figure S1 (a)

## Slide 3
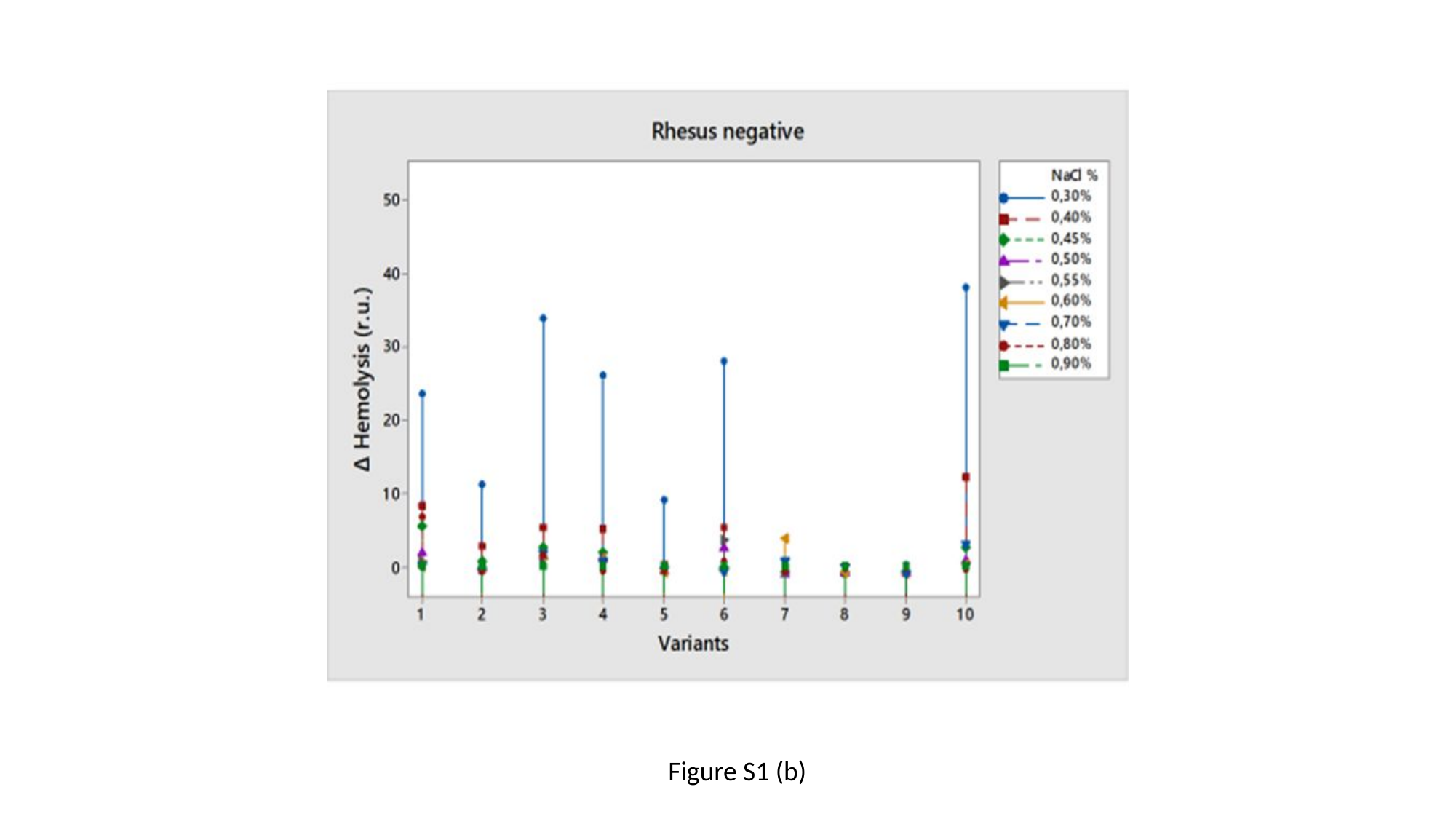

Figure S1 (b)

## Slide 4
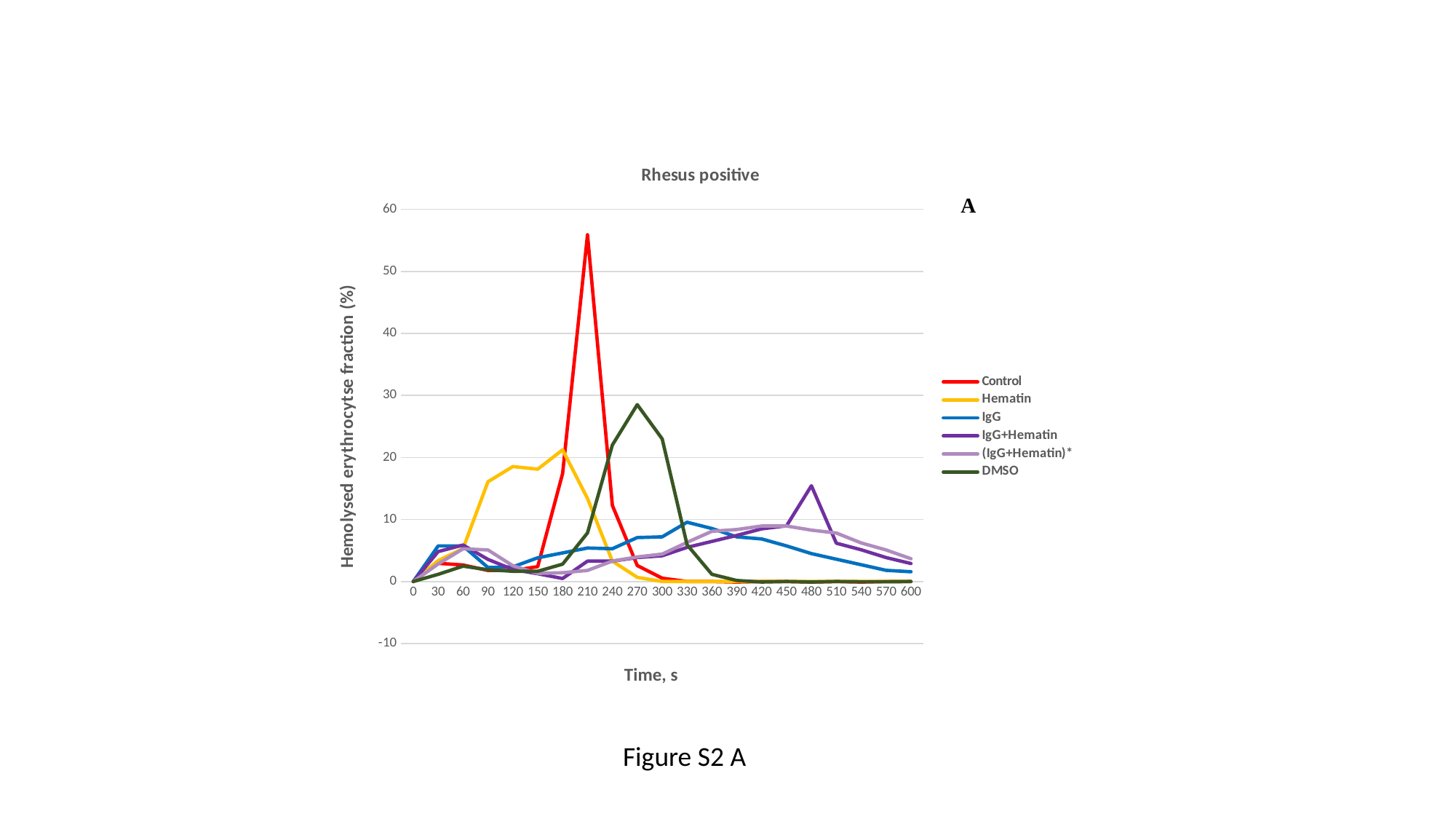

### Chart: Rhesus positive
| Category | Control | Hematin | IgG | IgG+Hematin | (IgG+Hematin)* | DMSO |
|---|---|---|---|---|---|---|
| 0 | 0.0 | 0.0 | 0.0 | 0.0 | 0.0 | 0.0 |
| 30 | 2.933333 | 3.36984 | 5.736783 | 4.826255 | 2.824859 | 1.154163 |
| 60 | 2.666667 | 5.307498 | 5.736783 | 5.888031 | 5.27307 | 2.473207 |
| 90 | 1.777778 | 16.09099 | 2.249719 | 3.571429 | 5.084746 | 1.896125 |
| 120 | 1.777778 | 18.53412 | 2.362205 | 1.930502 | 2.542373 | 1.648805 |
| 150 | 2.4 | 18.11289 | 3.824522 | 1.254826 | 1.318267 | 1.648805 |
| 180 | 17.42222 | 21.22999 | 4.611924 | 0.482625 | 1.412429 | 2.802968 |
| 210 | 55.91111 | 13.39511 | 5.399325 | 3.281853 | 1.789077 | 7.831822 |
| 240 | 12.26667 | 3.285594 | 5.286839 | 3.281853 | 3.295669 | 22.01154 |
| 270 | 2.577778 | 0.673968 | 7.086614 | 3.861004 | 3.954802 | 28.52432 |
| 300 | 0.533333 | 0.0 | 7.1991 | 4.150579 | 4.425612 | 23.00082 |
| 330 | 0.0 | 0.0 | 9.561305 | 5.501931 | 6.308851 | 5.935697 |
| 360 | 0.0 | 0.0 | 8.548931 | 6.467181 | 8.097928 | 1.154163 |
| 390 | -0.08889 | 0.0 | 7.1991 | 7.432432 | 8.380414 | 0.16488 |
| 420 | 0.0 | 0.0 | 6.861642 | 8.494208 | 8.945386 | -0.08244 |
| 450 | 0.0 | 0.0 | 5.736783 | 8.976834 | 8.945386 | 0.0 |
| 480 | -0.08889 | 0.0 | 4.499438 | 15.44402 | 8.286252 | -0.08244 |
| 510 | 0.0 | 0.0 | 3.59955 | 6.177606 | 7.815443 | 0.0 |
| 540 | -0.08889 | 0.0 | 2.699663 | 5.11583 | 6.214689 | -0.04122 |
| 570 | 0.0 | 0.0 | 1.799775 | 3.861004 | 5.084746 | -0.04122 |
| 600 | 0.0 | 0.0 | 1.574803 | 2.895753 | 3.672316 | 0.0 | Figure S2 A

## Slide 5
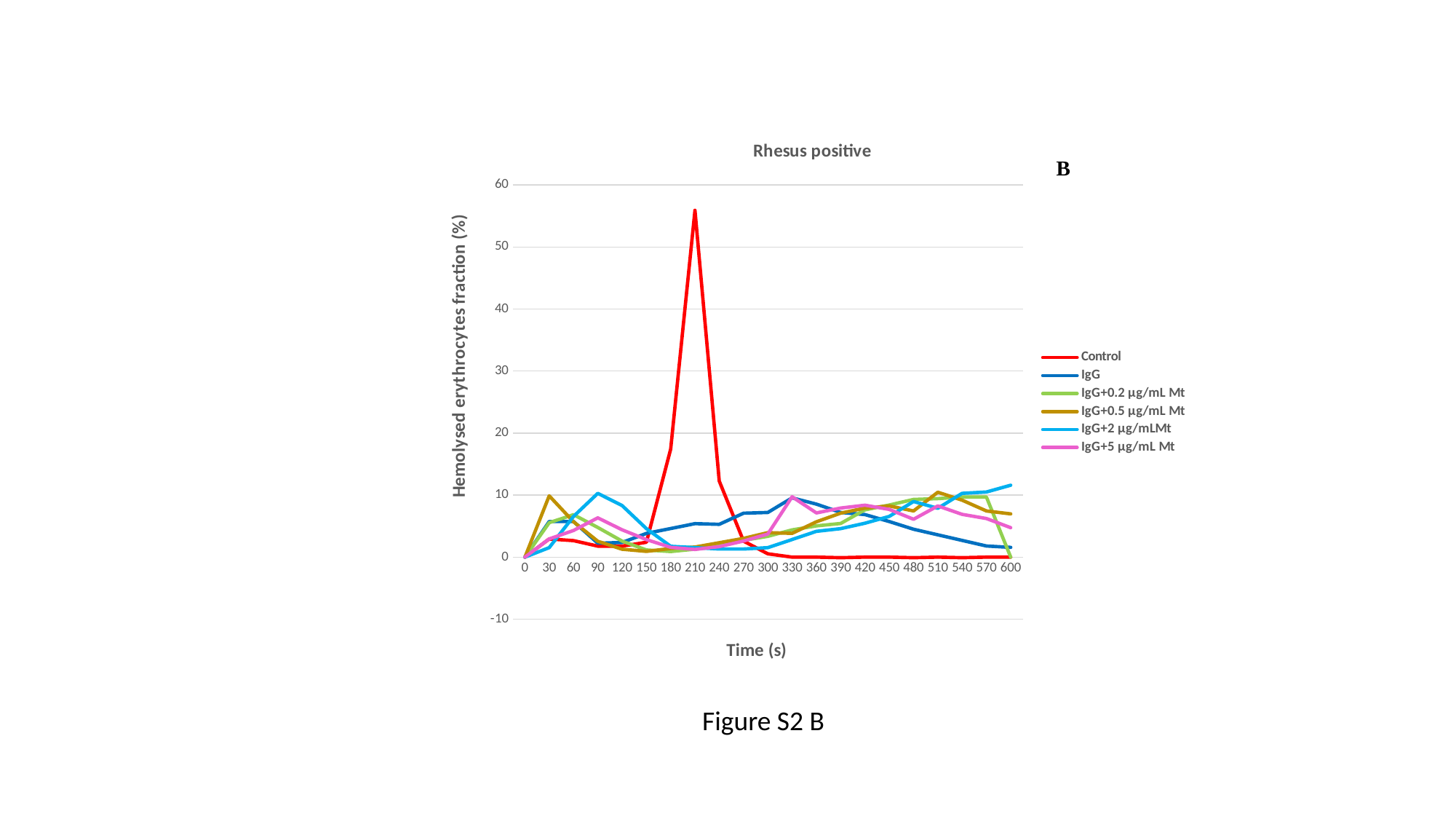

### Chart: Rhesus positive
| Category | Control | IgG | IgG+0.2 μg/mL Mt | IgG+0.5 μg/mL Mt | IgG+2 μg/mLMt | IgG+5 μg/mL Mt |
|---|---|---|---|---|---|---|
| 0 | 0.0 | 0.0 | 0.0 | 0.0 | 0.0 | 0.0 |
| 30 | 2.933333 | 5.736783 | 5.555556 | 9.883721 | 1.531729 | 2.941176 |
| 60 | 2.666667 | 5.736783 | 6.847545 | 5.697674 | 6.564551 | 4.298643 |
| 90 | 1.777778 | 2.249719 | 4.780362 | 2.55814 | 10.28446 | 6.334842 |
| 120 | 1.777778 | 2.362205 | 2.583979 | 1.27907 | 8.315098 | 4.411765 |
| 150 | 2.4 | 3.824522 | 1.162791 | 0.930233 | 4.595186 | 2.828054 |
| 180 | 17.42222 | 4.611924 | 0.904393 | 1.395349 | 1.750547 | 1.58371 |
| 210 | 55.91111 | 5.399325 | 1.29199 | 1.627907 | 1.531729 | 1.244344 |
| 240 | 12.26667 | 5.286839 | 1.808786 | 2.325581 | 1.31291 | 1.696833 |
| 270 | 2.577778 | 7.086614 | 2.713178 | 3.023256 | 1.31291 | 2.60181 |
| 300 | 0.533333 | 7.1991 | 3.359173 | 3.953488 | 1.531729 | 3.733032 |
| 330 | 0.0 | 9.561305 | 4.392765 | 3.837209 | 2.844639 | 9.728507 |
| 360 | 0.0 | 8.548931 | 5.03876 | 5.697674 | 4.157549 | 7.126697 |
| 390 | -0.08889 | 7.1991 | 5.426357 | 7.093023 | 4.595186 | 7.918552 |
| 420 | 0.0 | 6.861642 | 7.622739 | 7.906977 | 5.47046 | 8.371041 |
| 450 | 0.0 | 5.736783 | 8.397933 | 8.255814 | 6.564551 | 7.692308 |
| 480 | -0.08889 | 4.499438 | 9.302326 | 7.44186 | 8.971554 | 6.108597 |
| 510 | 0.0 | 3.59955 | 9.431525 | 10.46512 | 7.877462 | 8.257919 |
| 540 | -0.08889 | 2.699663 | 9.689922 | 9.186047 | 10.28446 | 6.900452 |
| 570 | 0.0 | 1.799775 | 9.689922 | 7.44186 | 10.50328 | 6.221719 |
| 600 | 0.0 | 1.574803 | 0.0 | 6.976744 | 11.59737 | 4.751131 | Figure S2 B

## Slide 6
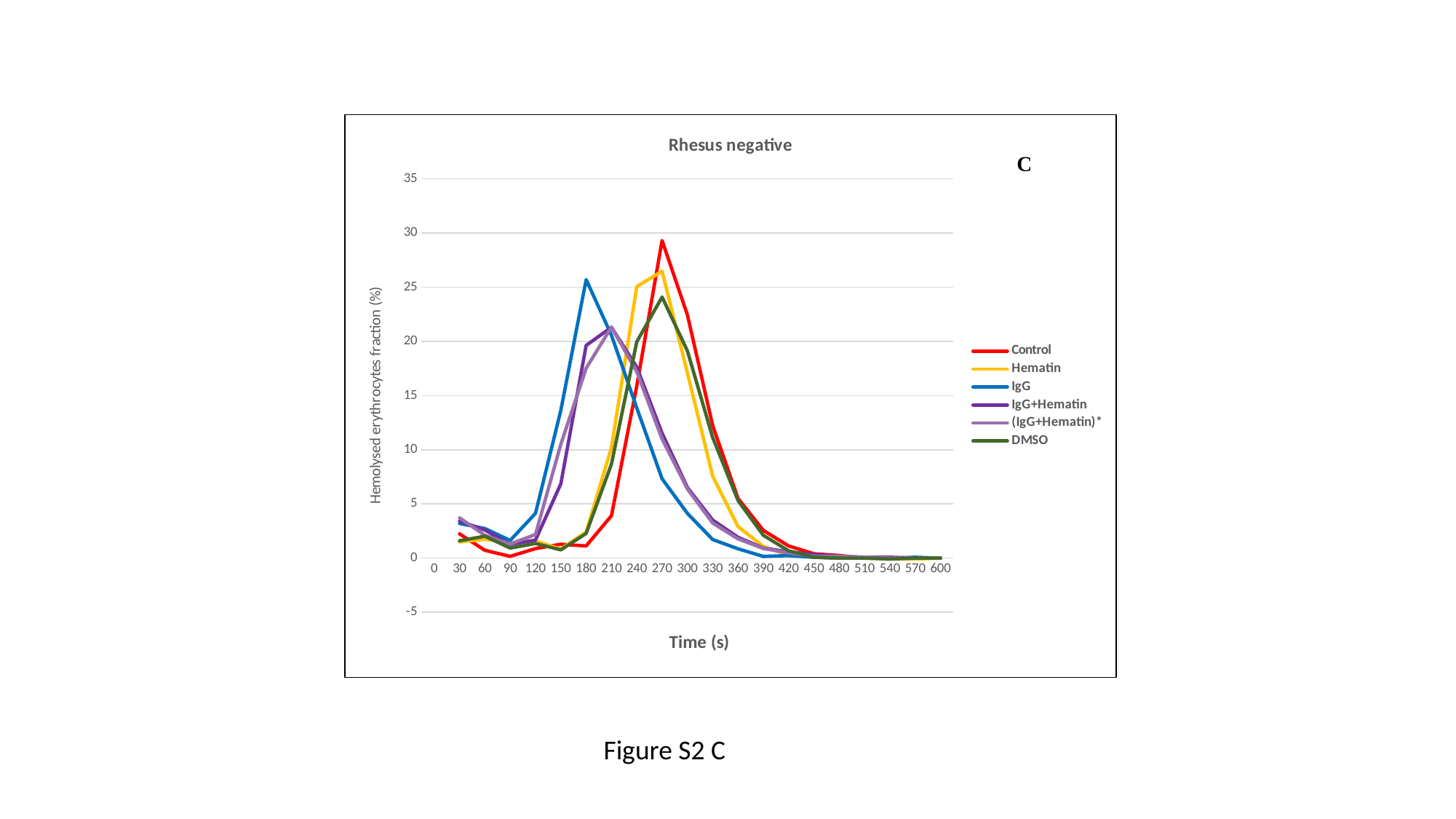

### Chart: Rhesus negative
| Category | Control | Hematin | IgG | IgG+Hematin | (IgG+Hematin)* | DMSO |
|---|---|---|---|---|---|---|
| 0 | None | None | None | None | None | None |
| 30 | 2.236422 | 1.493776 | 3.193146 | 3.42523 | 3.71567 | 1.600674 |
| 60 | 0.71885 | 1.742739 | 2.725857 | 2.589808 | 2.100162 | 2.021904 |
| 90 | 0.159744 | 1.410788 | 1.635514 | 1.253133 | 1.292407 | 0.926706 |
| 120 | 0.878594 | 1.576763 | 4.127726 | 1.670844 | 2.180937 | 1.347936 |
| 150 | 1.277955 | 0.829876 | 13.62928 | 6.850459 | 10.50081 | 0.758214 |
| 180 | 1.118211 | 2.406639 | 25.70093 | 19.63241 | 17.52827 | 2.274642 |
| 210 | 3.913738 | 10.20747 | 20.56075 | 21.30326 | 21.32472 | 8.677338 |
| 240 | 15.89457 | 25.06224 | 13.86293 | 17.6274 | 17.20517 | 19.9663 |
| 270 | 29.3131 | 26.47303 | 7.320872 | 11.52882 | 10.98546 | 24.09436 |
| 300 | 22.44409 | 17.09544 | 4.127726 | 6.516291 | 6.38126 | 19.12384 |
| 330 | 12.22045 | 7.551867 | 1.713396 | 3.508772 | 3.231018 | 11.12047 |
| 360 | 5.511182 | 2.904564 | 0.856698 | 1.92147 | 1.77706 | 5.307498 |
| 390 | 2.555911 | 1.078838 | 0.155763 | 0.918964 | 0.88853 | 2.10615 |
| 420 | 1.118211 | 0.248963 | 0.233645 | 0.584795 | 0.484653 | 0.673968 |
| 450 | 0.399361 | 0.082988 | 0.077882 | 0.334169 | 0.161551 | 0.084246 |
| 480 | 0.239617 | 0.0 | 0.0 | 0.167084 | 0.161551 | 0.0 |
| 510 | 0.0 | 0.0 | 0.0 | 0.083542 | 0.080775 | 0.0 |
| 540 | 0.0 | -0.08299 | 0.0 | 0.083542 | 0.0 | -0.08425 |
| 570 | 0.0 | -0.08299 | 0.077882 | 0.0 | 0.0 | 0.0 |
| 600 | 0.0 | 0.0 | 0.0 | 0.0 | 0.0 | 0.0 |Figure S2 C

## Slide 7
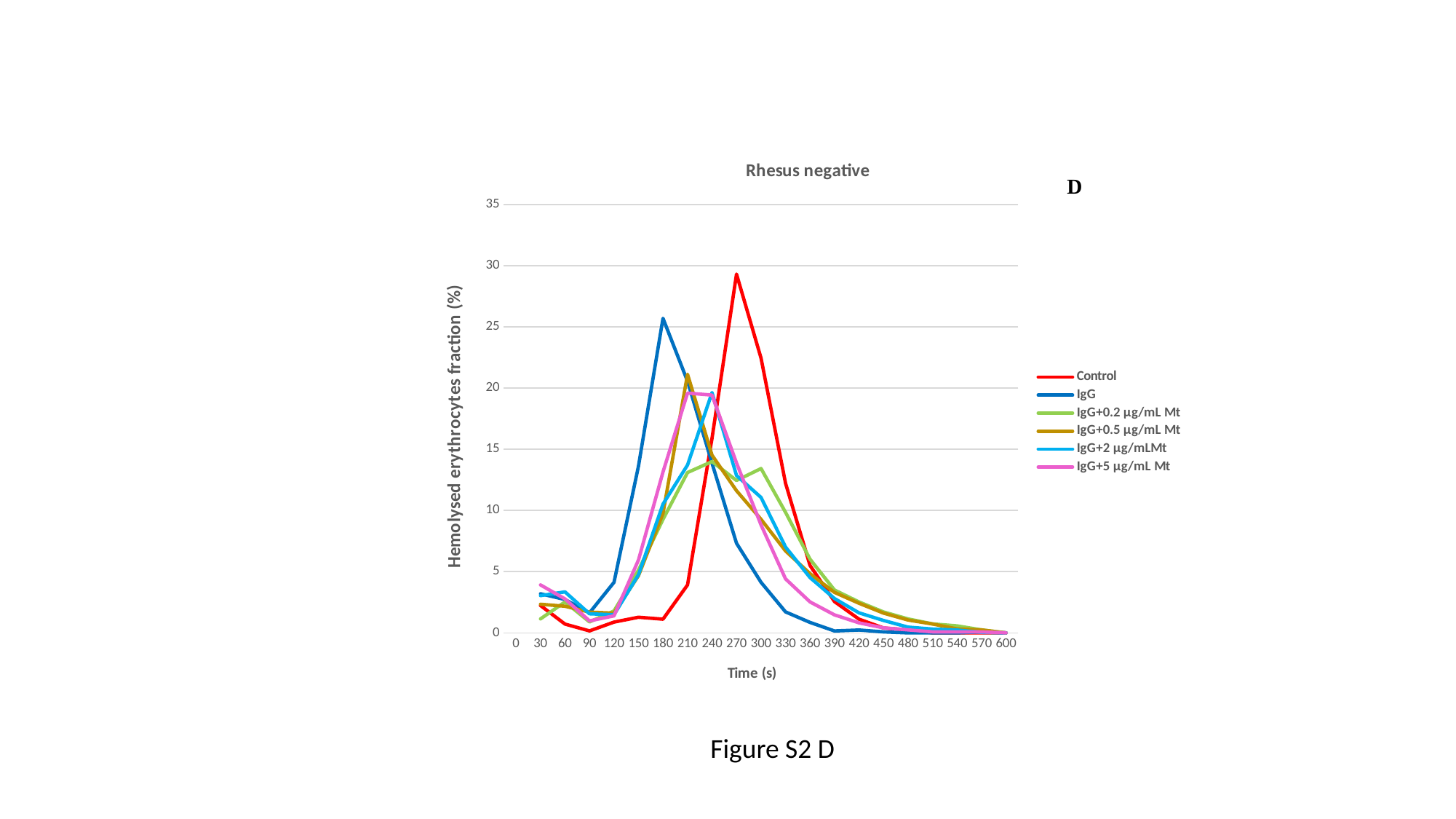

### Chart: Rhesus negative
| Category | Control | IgG | IgG+0.2 μg/mL Mt | IgG+0.5 μg/mL Mt | IgG+2 μg/mLMt | IgG+5 μg/mL Mt |
|---|---|---|---|---|---|---|
| 0 | None | None | None | None | None | None |
| 30 | 2.236422 | 3.193146 | 1.139138 | 2.33871 | 3.038566 | 3.918367 |
| 60 | 0.71885 | 2.725857 | 2.522376 | 2.177419 | 3.350214 | 2.77551 |
| 90 | 0.159744 | 1.635514 | 0.895037 | 1.693548 | 1.558239 | 0.979592 |
| 120 | 0.878594 | 4.127726 | 1.790073 | 1.612903 | 1.480327 | 1.387755 |
| 150 | 1.277955 | 13.62928 | 5.126119 | 4.677419 | 4.75263 | 5.959184 |
| 180 | 1.118211 | 25.70093 | 9.275834 | 9.758065 | 10.51811 | 13.14286 |
| 210 | 3.913738 | 20.56075 | 13.10008 | 21.12903 | 13.7125 | 19.59184 |
| 240 | 15.89457 | 13.86293 | 13.99512 | 14.51613 | 19.63381 | 19.42857 |
| 270 | 29.3131 | 7.320872 | 12.44915 | 11.6129 | 12.85547 | 13.87755 |
| 300 | 22.44409 | 4.127726 | 13.42555 | 9.274194 | 11.0635 | 8.816327 |
| 330 | 12.22045 | 1.713396 | 9.845403 | 6.693548 | 7.012076 | 4.408163 |
| 360 | 5.511182 | 0.856698 | 6.021155 | 4.83871 | 4.518894 | 2.530612 |
| 390 | 2.555911 | 0.155763 | 3.498779 | 3.306452 | 2.804831 | 1.469388 |
| 420 | 1.118211 | 0.233645 | 2.522376 | 2.419355 | 1.636151 | 0.816327 |
| 450 | 0.399361 | 0.077882 | 1.708706 | 1.612903 | 1.012855 | 0.408163 |
| 480 | 0.239617 | 0.0 | 1.139138 | 1.048387 | 0.467472 | 0.244898 |
| 510 | 0.0 | 0.0 | 0.732303 | 0.725806 | 0.311648 | 0.081633 |
| 540 | 0.0 | 0.0 | 0.569569 | 0.322581 | 0.233736 | 0.081633 |
| 570 | 0.0 | 0.077882 | 0.244101 | 0.241935 | 0.038956 | 0.081633 |
| 600 | 0.0 | 0.0 | 0.0 | 0.0 | 0.0 | 0.0 | Figure S2 D

## Slide 8
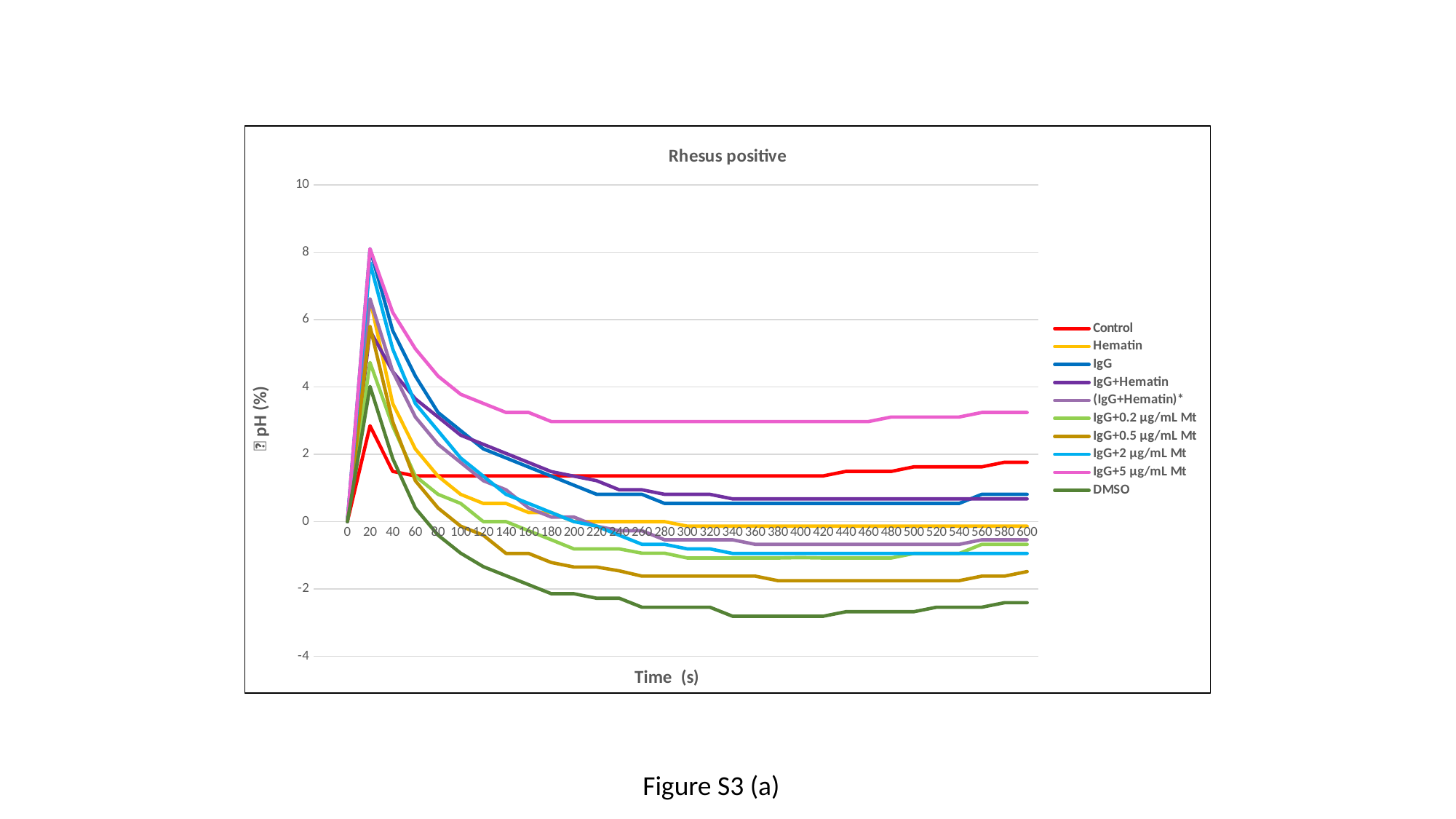

### Chart: Rhesus positive
| Category | Control | Hematin | IgG | IgG+Hematin | (IgG+Hematin)* | IgG+0.2 μg/mL Mt | IgG+0.5 μg/mL Mt | IgG+2 μg/mL Mt | IgG+5 μg/mL Mt | DMSO |
|---|---|---|---|---|---|---|---|---|---|---|
| | None | None | None | None | None | None | None | None | None | None |
| 0 | 0.0 | 0.0 | 0.0 | 0.0 | 0.0 | 0.0 | 0.0 | 0.0 | 0.0 | 0.0 |
| 20 | 2.849389 | 6.612686 | 8.108108 | 5.675676 | 6.621622 | 4.72973 | 5.802969 | 7.692308 | 8.108108 | 4.016064 |
| 40 | 1.492537 | 3.508772 | 5.675676 | 4.459459 | 4.459459 | 2.837838 | 2.968961 | 5.128205 | 6.216216 | 1.874163 |
| 60 | 1.356852 | 2.159244 | 4.324324 | 3.648649 | 3.108108 | 1.351351 | 1.214575 | 3.508772 | 5.135135 | 0.401606 |
| 80 | 1.356852 | 1.349528 | 3.243243 | 3.108108 | 2.297297 | 0.810811 | 0.404858 | 2.699055 | 4.324324 | -0.40161 |
| 100 | 1.356852 | 0.809717 | 2.702703 | 2.567568 | 1.756757 | 0.540541 | -0.13495 | 1.889339 | 3.783784 | -0.93708 |
| 120 | 1.356852 | 0.539811 | 2.162162 | 2.297297 | 1.216216 | 0.0 | -0.40486 | 1.349528 | 3.513514 | -1.33869 |
| 140 | 1.356852 | 0.539811 | 1.891892 | 2.027027 | 0.945946 | 0.0 | -0.94467 | 0.809717 | 3.243243 | -1.60643 |
| 160 | 1.356852 | 0.269906 | 1.621622 | 1.756757 | 0.405405 | -0.27027 | -0.94467 | 0.539811 | 3.243243 | -1.87416 |
| 180 | 1.356852 | 0.269906 | 1.351351 | 1.486486 | 0.135135 | -0.54054 | -1.21457 | 0.269906 | 2.972973 | -2.1419 |
| 200 | 1.356852 | 0.0 | 1.081081 | 1.351351 | 0.135135 | -0.81081 | -1.34953 | 0.0 | 2.972973 | -2.1419 |
| 220 | 1.356852 | 0.0 | 0.810811 | 1.216216 | -0.13514 | -0.81081 | -1.34953 | -0.13495 | 2.972973 | -2.27577 |
| 240 | 1.356852 | 0.0 | 0.810811 | 0.945946 | -0.27027 | -0.81081 | -1.46277 | -0.40486 | 2.972973 | -2.27577 |
| 260 | 1.356852 | 0.0 | 0.810811 | 0.945946 | -0.27027 | -0.93708 | -1.61943 | -0.67476 | 2.972973 | -2.54351 |
| 280 | 1.356852 | 0.0 | 0.540541 | 0.810811 | -0.54054 | -0.93708 | -1.61943 | -0.67476 | 2.972973 | -2.54351 |
| 300 | 1.356852 | -0.13495 | 0.540541 | 0.810811 | -0.54054 | -1.08108 | -1.61943 | -0.80972 | 2.972973 | -2.54351 |
| 320 | 1.356852 | -0.13495 | 0.540541 | 0.810811 | -0.54054 | -1.08108 | -1.61943 | -0.80972 | 2.972973 | -2.54351 |
| 340 | 1.356852 | -0.13495 | 0.540541 | 0.675676 | -0.54054 | -1.08108 | -1.61943 | -0.94467 | 2.972973 | -2.81124 |
| 360 | 1.356852 | -0.13495 | 0.540541 | 0.675676 | -0.67568 | -1.08108 | -1.61943 | -0.94467 | 2.972973 | -2.81124 |
| 380 | 1.356852 | -0.13495 | 0.540541 | 0.675676 | -0.67568 | -1.08108 | -1.75439 | -0.94467 | 2.972973 | -2.81124 |
| 400 | 1.356852 | -0.13495 | 0.540541 | 0.675676 | -0.67568 | -1.06952 | -1.75439 | -0.94467 | 2.972973 | -2.81124 |
| 420 | 1.356852 | -0.13495 | 0.540541 | 0.675676 | -0.67568 | -1.08108 | -1.75439 | -0.94467 | 2.972973 | -2.81124 |
| 440 | 1.492537 | -0.13495 | 0.540541 | 0.675676 | -0.67568 | -1.08108 | -1.75439 | -0.94467 | 2.972973 | -2.67738 |
| 460 | 1.492537 | -0.13495 | 0.540541 | 0.675676 | -0.67568 | -1.08108 | -1.75439 | -0.94467 | 2.972973 | -2.67738 |
| 480 | 1.492537 | -0.13495 | 0.540541 | 0.675676 | -0.67568 | -1.08108 | -1.75439 | -0.94467 | 3.108108 | -2.67738 |
| 500 | 1.628223 | -0.13495 | 0.540541 | 0.675676 | -0.67568 | -0.94595 | -1.75439 | -0.94467 | 3.108108 | -2.67738 |
| 520 | 1.628223 | -0.13495 | 0.540541 | 0.675676 | -0.67568 | -0.94595 | -1.75439 | -0.94467 | 3.108108 | -2.54351 |
| 540 | 1.628223 | -0.13495 | 0.540541 | 0.675676 | -0.67568 | -0.94595 | -1.75439 | -0.94467 | 3.108108 | -2.54351 |
| 560 | 1.628223 | -0.13495 | 0.810811 | 0.675676 | -0.54054 | -0.67568 | -1.61943 | -0.94467 | 3.243243 | -2.54351 |
| 580 | 1.763908 | -0.13495 | 0.810811 | 0.675676 | -0.54054 | -0.67568 | -1.61943 | -0.94467 | 3.243243 | -2.40964 |
| 600 | 1.763908 | -0.13495 | 0.810811 | 0.675676 | -0.54054 | -0.67568 | -1.48448 | -0.94467 | 3.243243 | -2.40964 | Figure S3 (a)

## Slide 9
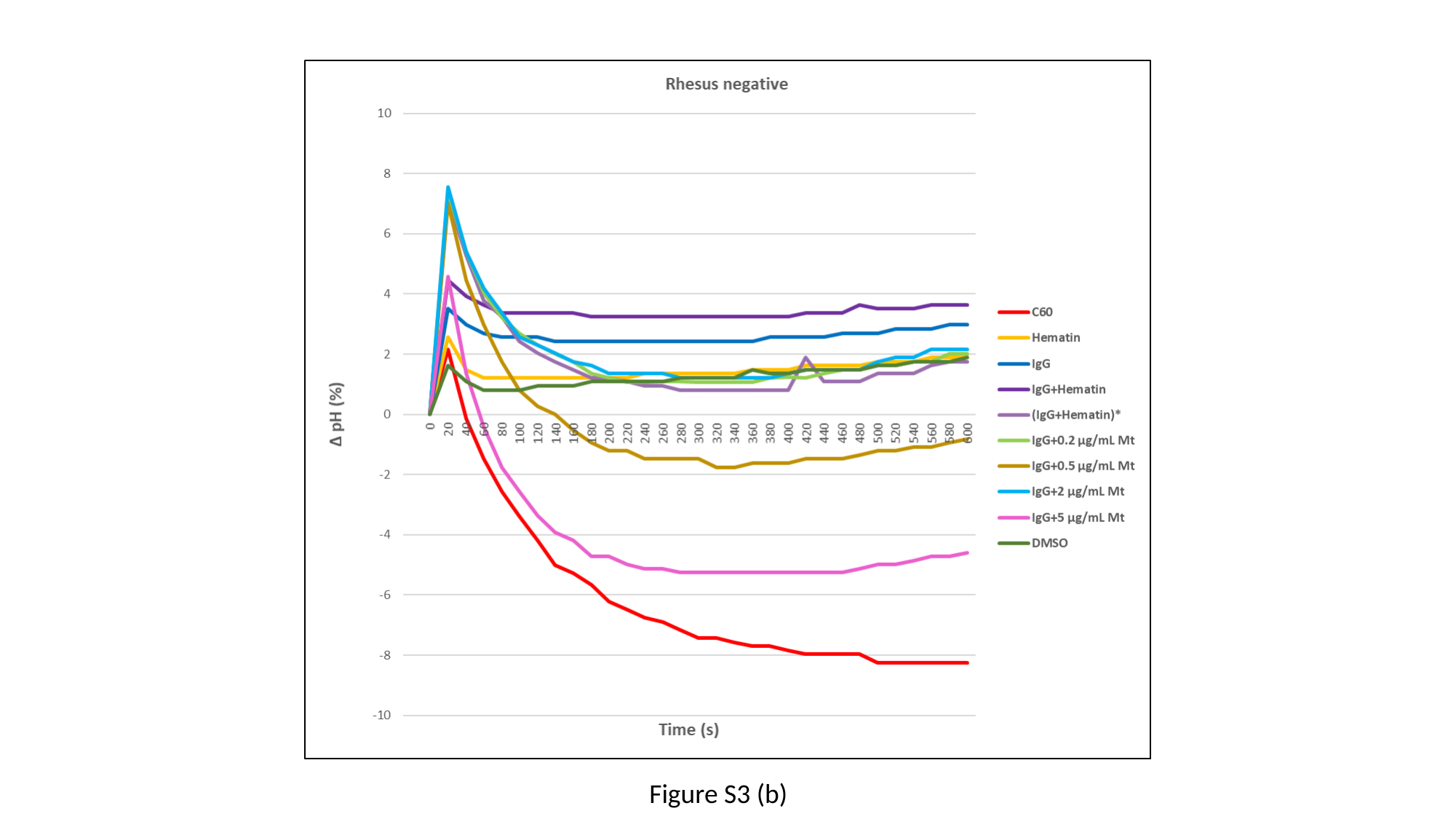

Figure S3 (b)
